# Time- and Dose-Dependent Effects of Irradiation on Endothelial and Tumor Endothelial Cells: Transcriptional, Molecular, and Functional Changes Driving Activation In Vitro and In Vivo

**DOI:** 10.3390/cancers17172842

**Published:** 2025-08-29

**Authors:** Iva Santek, Gregor Sersa, Bostjan Markelc

**Affiliations:** 1 Department of Experimental Oncology, Institute of Oncology Ljubljana, Zaloska cesta 2, 1000 Ljubljana, Slovenia; isantek@onko-i.si (I.S.); gsersa@onko-i.si (G.S.); 2 Faculty of Medicine, University of Ljubljana, Vrazov trg 2, 1000 Ljubljana, Slovenia; 3 Faculty of Health Sciences, University of Ljubljana, Zdravstvena pot 5, 1000 Ljubljana, Slovenia; 4 Biotechnical Faculty, University of Ljubljana, Jamnikarjeva ulica 101, 1000 Ljubljana, Slovenia

**Keywords:** endothelial cell activation, irradiation, radiotherapy, tumor vasculature

## Abstract

**Background:** Irradiation (IR) targets cancer cells, but also the tumor microenvironment, including the tumor’s blood vessels. In addition to tumor endothelial cell (TEC) apoptosis, IR can lead to TEC activation, potentially increasing immune cell infiltration. However, the changes underlying the IR-induced activation of endothelial cells (ECs) are poorly understood. This study investigated dose- and time-dependent molecular and functional responses of murine and human EC lines to IR in vitro and TECs in vivo in murine tumor models of colorectal carcinoma. **Methods:** HUVEC, EA.hy926, and Hulec5a, as well as murine bEND.3, 2H11, and SVEC4-10 EC lines, were irradiated with single doses of 2–10 Gy. EC proliferation and survival after IR were assessed by staining all nuclei (Hoechst 33342) and dead cells (propidium iodide) every 24 h for 5 days using the Cytation 1 Cell Imaging Multi-Mode Reader. RNA sequencing analysis of HUVECs irradiated with 2 Gy and 5 Gy at 24 h and 72 h after IR was conducted, focusing on processes related to EC activation. To validate the RNA sequencing results, immunofluorescence staining for proteins related to EC activation, including Stimulator of Interferon Response cGAMP Interactor 1 (STING), Nuclear factor kappa B (NF-κβ), and Vascular cell adhesion molecule 1 (VCAM-1), was performed. To validate the in vitro results, the response of TEC in vivo was analyzed using publicly available RNA sequencing data of TECs isolated from MC38 colon carcinoma irradiated with a single dose of 15 Gy. Finally, murine CT26 colon carcinoma tumors were immunofluorescently stained for STING and NF-κβ 24 and 48 h after IR with a clinically relevant fractionated regimen of 5 × 5 Gy. **Results:** Doses of 2, 4, 6, 8, and 10 Gy led to a dose-dependent decrease in proliferation and increased death of ECs. RNA sequencing analysis showed that the effects on the transcriptome of HUVECs were most pronounced 72 h after IR with 5 Gy, with 1014 genes (661 down-regulated and 353 up-regulated) being significantly differentially expressed. Irradiation with 5 Gy resulted in HUVEC activation, with up-regulation of the immune system and extracellular matrix genes, such as *STING1* (log_2_FC = 0.81) and *SELE* (log_2_FC = 1.09), respectively; and down-regulation of cell cycle markers. Furthermore, IR led to the up-regulation of immune response- and extracellular matrix (ECM)-associated signaling pathways, including NF-κβ signaling and ECM–receptor interaction, which was also observed in the transcriptome of irradiated murine TECs in vivo. This was confirmed at the protein level with higher expressions of the EC activation-associated proteins STING, NF-κβ, and VCAM-1 in irradiated HUVECs and irradiated TECs in vivo. **Conclusions:** IR induces changes in ECs and TECs, supporting their activation in dose- and time-dependent manners, potentially contributing to the anti-tumor immune response, which may potentially increase the infiltration of immune cells into the tumor and thus, improve the overall efficacy of RT, especially in combination with immune checkpoint inhibitors.

## Simple Summary

Abnormal tumor vasculature is marked by inadequate blood flow and oxygen delivery, causing the formation of hypoxic areas, resistant to radiotherapy (RT). Irradiation (IR) affects not only cancer cells but also the tumor microenvironment, including tumor blood vessels. Intriguingly, besides triggering the apoptosis of tumor endothelial cells (TECs), IR can also lead to vascular normalization/remodeling or TEC activation, potentially alleviating hypoxia and facilitating immune cell infiltration. However, the role of IR-induced alterations of TECs and endothelial cells (ECs) on the response to RT remains poorly understood. Therefore, this study aimed to clarify how vasculature responds to IR, focusing on EC and TEC activation. Results showed a reduction in EC proliferation and survival after IR in vitro, in dose- and time-dependent manners. Importantly, RNA sequencing results showed down-regulation of cell cycle and up-regulation of signaling pathways related to EC activation in irradiated HUVECs, which was further confirmed on a protein level and in vivo in murine TECs. Optimizing radiotherapy (RT) to take advantage of the potentially increased infiltration of immune cells into the tumor through activated TECs could improve the overall efficacy of RT, especially in combination with immune checkpoint inhibitors.

## 1. Introduction

Radiotherapy (RT) is one of the most used anti-tumor therapies [1,2,3]. In colorectal (CRC) cancer, which is the second most prevalent cancer in women and third in men, preoperative RT is often used to reduce local recurrence after surgery. However, even in this setting, some patients still relapse [4]. Irradiation (IR) does not only affect cancer cells but also the tumor microenvironment (TME), including tumor blood vessels [5,6]. The tumor vasculature is functionally and structurally abnormal, resulting in poor perfusion and therefore, inadequate blood flow and oxygen delivery. Therefore, the outcome of RT is also determined by changes occurring in the tumor vasculature; however, the response of the tumor endothelium to RT is poorly understood.

Irradiation (IR) affects the survival, proliferation, and physiological properties of tumor endothelial cells (TECs) in a dose- and time-dependent manner [7]. IR can induce the apoptosis of TECs, with the proliferating endothelium being more susceptible than the quiescent endothelium [6]. Despite TEC death after IR with single doses below 15 Gy or daily fractionated doses, Kaeppler, J.R. et al. showed no vascular destruction and a minimal effect of IR on the vascular structure [7]. However, whether survived ECs are still capable of proliferation and thus, revascularization of the post-irradiated tumors remains an unanswered question [6]. Furthermore, doses higher than 2 Gy can lead to endothelium activation, during which endothelial cells (ECs) switch from an anti-inflammatory to a pro-inflammatory phenotype [6,8,9]. The underlying mechanism for the activation of tumor endothelium remains unknown; however, accumulation of DNA in their cytosol after IR activating the cyclic GMP-AMP synthase (cGAS)–stimulator of interferon genes (STING) pathway, as is the case in tumor cells and macrophages, could be one of the reasons [10]. Activated endothelium is characterized by overexpression of adhesion molecules, such as vascular cell adhesion molecule 1 (VCAM-1), intercellular adhesion molecule 1 (ICAM-1), platelet and endothelial cell adhesion molecule 1 (PECAM-1), and E-selectin, partially due to IR- and ROS-activated nuclear factor kappa B (NF-κB), and pro-inflammatory chemokines and cytokines, including tumor necrosis factor alpha (TNFα) and interleukin 6 (IL-6) [11]. This multistep process contributes to the recruitment and infiltration of immune cells such as CD8+ cytotoxic T cells, which directly kill tumor cells, or CD4+ helper T cells, which augment the activation of cytotoxic lymphocytes by enhancing inflammation [10,11]. However, the exact crosstalk between IR-activated tumor ECs and immune cells leading to the augmented immune cell infiltration into tumors is yet to be elucidated.

Previous in vitro research showed that irradiated ECs undergo apoptosis and cellular senescence, which is time- and dose-dependent [12,13,14,15]. While single or fractionated IR with low doses (0.125, 0.25, and 0.5 Gy) does not affect EA.hy926 or HUVEC survival or apoptosis, higher doses, 4 Gy–10 Gy, caused radiation-induced DNA damage, inducing cellular senescence and/or apoptosis [16,17,18,19]. This is mainly due to the increased expression of senescence-associated genes, such as β-galactosidase, insulin-like growth factor binding protein 5 (*IGFBP5*), Jagged Canonical Notch Ligand 1 (*JAG1*), plasminogen activator (*PLAT*), and Sprouty homolog 4 (*SPRY4*) [19].

IR also induces changes related to the extracellular matrix (ECM) and immune activation in ECs in vitro, thus supporting endothelium activation. Hallahan et al. irradiated HUVECs with doses ranging from 0.5 to 50 Gy and followed the expression of adhesion molecules. They observed induced expressions of E-selectin and ICAM-1 in a dose- and time-dependent manner [20,21,22]. This was also confirmed in irradiated HMEC and EA.hy926 [23]. Another study showed that IR of HUVECs with 2 or 4 Gy led to the elevated permeability of endothelial monolayers and transendothelial migration of tumor cells due to the degradation of Vascular Endothelial Cadherin (VE-Cad), as a consequence of up-regulation of ADAM Metallopeptidase Domain 10 (ADAM10) [24]. Additionally, latest studies have reported that IR with a single dose of 10 Gy up-regulates endothelial-to-mesenchymal transition, characterized by the loss of cell–cell junctions and elevated cell motility, in irradiated HLMVECs [12]. The effect of IR on ECs in vitro is also characterized by ECs switching from an anti- to pro-inflammatory phenotype. This was observed after IR exposure to low and high IR doses, where irradiated HUVECs showed a significant increase in the production of reactive oxygen species (ROS) and NF-κβ activation and expression of pro-inflammatory cytokines, such as *IL-6* and *IL-8* [17,23]. Although recent studies suggested that the transcriptional response of ECs to IR are mediated by adenosine triphosphate (ATP) and adenosine diphosphate (ADP) signaling, more studies are required to confirm if this is a general phenomenon [25].

However, a detailed transcriptomic analysis of the molecular changes in the effects of IR on ECs in vitro and how these are related to EC activation has not yet been performed. Therefore, the aim of this study was to determine the dose- and time- dependent functional, molecular, and transcriptional changes in murine and human ECs and murine TECs after IR, with specific emphasis on EC activation.

## 2. Materials and Methods

### 2.1. EC Culturing

All EC lines used were purchased from the American Type Culture Collection (ATCC), maintained at 37 °C in a humidified atmosphere containing 5% CO_2_ and routinely tested for mycoplasma using the MycoAlert™ Plus Mycoplasma Detection Kit (Lonza, Basel, Switzerland). The EA.hy926 (ATCC, cat# CRL-2922), 2H11 (ATCC, cat# CRL-2163), SVEC4-10 (ATCC, #CRL-2181), and bEnd.3 (ATCC, cat# CRL-2299) cell lines were cultured in Advanced DMEM medium (Gibco, Waltham, MA, USA) supplemented with 5% fetal bovine serum (FBS) (Gibco). Hulec5a (ATCC, cat# CRL-3244) cells were cultured in MCDB medium (Thermo Fisher Scientific, Waltham, MA, USA) supplemented with 10% FBS. Additionally, DMEM and MCDB were supplemented with 10 mM L-glutamine, 50 mg/mL gentamicin, and 100 U/mL penicillin. HUVECs (ATCC, cat# PCS-100-13) were cultured in EGM^TM^-2 MV Microvascular Endothelial Cell Growth Medium-2 BulletKit^TM^ (Lonza), with media changes every other day. All cell lines were cultured until they reached approximately 70% confluence. In the experiments, cells up to 10th passage were utilized. For experiments determining cell proliferation and survival after IR, 2000 cells/well were seeded in 100 µL of culture medium in a 96-well plate (Corning, New York, NY, USA), except for 2H11 cells, where 1000 cells/well were seeded. For RNA sequencing experiments, 1.3 × 10^5^ of HUVECs were seeded in 7 mL of culture medium in T25 flasks (VWR). The cells were cultured until a monolayer was formed and then irradiated.

### 2.2. EC Irradiation

IR was conducted with a Gulmay CP225 X-RAY generator with 0.55 mm copper and 1.8 mm aluminum filtering, operated at 200 kV, 9.2 mA, with a dose rate of 1.73 Gy/min in in vitro experiments and 1.92 Gy/min in in vivo experiments. Dosimetry of the irradiation setup was performed to determine the exact dose rate for in vitro and in vivo experiments. In the cell proliferation and survival experiments, cells were subjected to 2, 4, 6, 8, or 10 Gy of IR one day after seeding. For RNA sequencing in vitro, only doses of 2 or 5 Gy were delivered to the HUVECs. The selection of IR doses for RNA sequencing in vitro was based on the literature, with the 2 Gy dose chosen due to its capability to induce endothelium activation, and the 5 Gy dose corresponding to one fraction of the RT regimen used in clinics (5 × 5 Gy) [26]. In the in vivo experiments, IR was administered locally to subcutaneously growing CT26 colon carcinoma tumors in mice. IR was performed once the tumors reached a volume of 60–80 mm^3^. During IR treatment, mice were restrained in a lead shield that covered the entire mouse, leaving only the tumor exposed to IR, thereby preventing IR of the surrounding tissue and the rest of the mouse. In total, a fractionated IR dose of 5 × 5 Gy was delivered by exposing the tumors to 5 Gy once daily for five consecutive days. For in vivo RNA sequencing analysis, a publicly available dataset (GSE168481) [7,27] derived from TECs isolated from irradiated subcutaneously growing MC38 colon carcinomas was analyzed. Briefly, as Kaeppler et al. described, a single dose of 15 Gy of IR was delivered locally to tumors, while mice were anesthetized with isoflurane and positioned in a led shield, ensuring that only the tumors were exposed to IR [7]. X-ray IR was delivered using a Gulmay RS320 IR system, operated at 300 kV, with a dose rate of 2.25 Gy per minute [27].

### 2.3. Determination of Cell Proliferation and Survival

Control (non-irradiated) cells and cells irradiated with a single dose of 2–10 Gy were cultured under standard conditions with media changes every second day. Immediately before IR treatment (0 h) and at different timepoints (24, 48, 72, and 96 h) after IR, 10 µL of phosphate-buffered saline (PBS) solution containing Hoechst 33342 (30 µg/mL, Trihydrochloride, Trihydrate, Thermo Fisher Scientific) and propidium iodide (PI, 10 µg/mL, Sigma Aldrich, St. Louis, MO, USA) were added to the cells, followed by one-hour incubation at 37 °C in a 5% CO_2_ humidified incubator. The number of live nuclei (Hoechst 33342 positive) and the number of dead cells (PI positive) were quantified using a Cytation 1 Cell Imaging Multi-Mode Reader (BioTek, Winooski, VT, USA). Relative in proliferation was calculated by comparing the count of Hoechst-positive nuclei at different timepoints after IR, with the count of Hoechst-positive nuclei before IR (t = 0 h). To quantify the EC death rate after IR, we compared the count of PI-positive nuclei at each time interval after IR with a number of PI-positive nuclei in non-irradiated ECs at corresponding timepoints. To assess the variation in doubling time between different IR regimens, we calculated the relative change in doubling time for each IR dose, derived as the doubling time after IR divided by the doubling time of the control samples, where doubling times were calculated using the Malthusian exponential growth model [26]. A comparison of radiosensitivity between cell lines was determined based on the relative change in proliferation compared with the control, which was calculated as the ratio of relative change in proliferation after IR at a certain timepoint and the relative change in proliferation of non-irradiated cells at the same timepoint.

### 2.4. Extraction of Total RNA and Messenger RNA (mRNA) Sequencing

To assess IR-induced changes in HUVEC transcriptome, five independent biological replicates (*n* = 5 per group) of HUVECs were used. Total RNA was extracted from cell passages ranging from p4 to p10 using the Total RNA Kit peqGOLD (VWR, West Chester, PA, USA) according to the manufacturer’s instructions. The purity of extracted RNA was measured spectrophotometrically using A260/A280 and A260/A230 ratios with a Cytation 1 Cell Imaging Multi-Mode Reader. RNA quality was assessed with the RNA Assay for LabChip^®^ GX Touch (PerkinElmer, Waltham, MA, USA) according to the manufacturer’s protocol. Samples with an RNA Quality Score above 9 were sent to Novogene (UK) Company LTD (Cambridge, UK) for transcriptome sequencing, which was conducted using the Illumina platform. To investigate the effect of IR on the activation of TECs, we performed an RNA sequencing analysis of a publicly available dataset arising from TEC FACS, sorted from MC38 murine colon carcinoma tumors irradiated with a single dose of 15 Gy (*n* = 5 per group, GSE168481) [7,27].

### 2.5. RNA Sequencing Analysis

Removal of adapters, data filtering, and data quality control; alignment to a reference genome (human assembly GRCh38/hg38); and estimation of transcript abundance was performed by Novogene (UK) Company LTD (Cambridge, UK). For differential gene expression analysis, R software was used (R Core Team, v4.4.1) [28]. Lowly expressed genes were filtered out, and data were normalized using the EdgeR (v4.4.0) R package [29]. For in vitro RNA sequencing data, batch effect removal was not performed as all samples were processed simultaneously, and principal component analysis (PCA) confirmed clustering according to experimental groups. For in vivo data, batch effects were accounted for by including batch as a covariate in the generalized linear model (GLM) design in EdgeR. Differential gene expression (DGE) analysis was also conducted with EdgeR, with *p*-values adjusted using Benjamini and Hochberg’s approach for controlling the false discovery rate (FDR = 0.05). Statistical significance was determined by applying a significance thresholds of FDR-adjusted *p*-value of <0.05 and a fold change (FC) of >1.5 for up-regulation or FC of <0.67 for down-regulation for in vitro experiments, and log_2_FC > 0.5 or log_2_FC < −1 for in vivo experiments. Functional Kyoto Encyclopedia of Genes and Genomes (KEGG) pathway analysis was performed using the clusterProfiler package (v4.16.0) [30,31]. Gene set enrichment analysis (GSEA) was performed using the GSA package (v1.3.3) [32,33]. Pathways with adjusted *p*-values of <0.05 were considered significantly enriched. In KEGG analysis, GeneRatio was calculated as the ratio of the number of up-regulated and down-regulated genes in each pathway, with GeneRatio > 1 representing up-regulation and GeneRatio < 1 representing down-regulation of a pathway. In GSEA, the direction of enrichment was determined by the Normalized Enrichment Score (NES), with positive values indicating up-regulation and negative values indicating down-regulation of the corresponding pathway.

### 2.6. Reverse Transcription and Quantitative Real-Time Polymerase Chain Reaction (qRT-PCR)

To validate the RNA sequencing results of the HUVEC transcriptome after 2 Gy or 5 Gy of IR, qRT-PCR was performed to assess the expression of selected key genes, including *SELE*, *TNFSF4*, and *CXCL12*. Reverse transcription was performed using the qScript™ Ultra SuperMix kit (Quantabio, Beverly, MA, USA) according to the manufacturer’s protocol. The resulting cDNA was used as a template for qRT-PCR, with 10 ng of cDNA used in each reaction. Pre-designed primers (Hs.PT.58.1165629 (*SELE*), Hs.PT.58.20738450 (*TNFSF4*), Hs.PT.58.28013422 (*CXCL12*), Hs.PT.39a.19639531 (*POLR2A*)), Integrated DNA Technologies, and FastGene^®^ IC Green 2x qPCR Universal Mix (Genetics) were utilized. Amplification was performed using QuantStudio™ 5 Real-Time PCR System (Thermo Fisher Scientific). Relative gene expression (fold change) was calculated using the 2^−∆∆Ct^ method, with *POLR2A* gene serving as the housekeeping control. Irradiated samples were normalized to the corresponding non-irradiated controls.

### 2.7. Immunofluorescence In Vitro

Immunofluorescence was used to evaluate the activation of Stimulator of Interferon Response CGAMP Interactor 1 (STING), NF-κβ, and VCAM-1 after IR. In total, 1.3 × 10^4^ HUVECs in 250 µL of media were seeded into each well of a removable 12-well Ibidi Chamber (Ibidi, Gräfelfing, Germany) and cultured under standard conditions for 6 days or until the cells reached a 100% confluent monolayer. The chambers were then irradiated with 2 and 5 Gy, except for the control chambers, and incubated for 24 or 72 h, followed by immunofluorescence staining. For each group, three biological replicates (each with two technical replicates) were stained. In all washing steps, cells were incubated twice for 5 min in Hanks’ Balanced Salt Solution (HBSS+/+, calcium, magnesium, no phenol red, Gibco, Thermo Fisher Scientific) or PBS, as stated. After the initial washing with HBSS+/+, to achieve membrane staining, cells were incubated in a 1 µg/mL wheat germ agglutinin (WGA) Alexa Fluor 647 conjugate solution (Thermo Fisher Scientific) in HBSS+/+ for 10 min at room temperature, followed by washing with HBSS+/+. Cells were then fixed in pre-warmed 4% paraformaldehyde (PFA; Alfa Aesar, Thermo Fisher Scientific) for 15 min at 37 °C and washed with HBSS+/+. To permeabilize the cells, Tween 20 in PBS was used in a 0.02% solution for STING and in a 0.5% solution for NF-κβ and VCAM-1 for 15 min at room temperature. Cells were washed with PBS and incubated in blocking buffer (0.01% Tween 20, 5% donkey serum, 22.52 mg/mL glycine in PBS) for 1 h at room temperature to prevent nonspecific binding. Primary antibodies for STING (Novus Biologicals, Centennial, CO, USA, #NBP2-24683), NF-κβ (Abcam, Cambridge, UK, #ab140751), and VCAM-1 (Abcam, #ab134047) were diluted in blocking buffer at a dilution of 1:200 and added to the cells, followed by overnight incubation at 4 °C. The next day, cells were washed three times with PBS for 5 min and incubated with the secondary antibody Alexa Fluor 488 (Jackson ImmunoResearch, West Grove, PA, USA, #711-545-155 and #703-545-155) diluted to 1:400 in PBS for 1 h at room temperature. To stain nuclei, cells were washed in PBS and incubated in PBS containing Hoechst 33342 (30 µg/mL, Thermo Fisher Scientific), followed by three washes in PBS. Afterwards, the removable silicone chambers (Ibidi) were removed, and slides were mounted with Prolong Glass Antifade Mountant (Thermo Fisher Scientific) for 3 days at room temperature and sealed with nail polish. Imaging was conducted with an LSM 800 confocal microscope (Carl Zeiss, Oberkochen, Germany) using a 63× oil immersion objective (NA 1.4). To excite Hoechst 33342, Alexa Fluor 488, and Alexa Fluor 647, excitation wavelengths of 405, 488, and 640 nm were used, respectively. The resultant emitted light was collected sequentially using a Gallium Arsenide Phosphide (GaAsP) detector, employing a variable dichroic and filters at specified wavelengths: 410–545 nm (Hoechst 33342), 488–545 nm (Alexa Fluor 488), and 645–700 nm (Alexa Fluor 647). Three or more random fields of view from three biological replicates (*n* = 3) per group were acquired. The acquired images were analyzed and visualized using Imaris software (v9.9.1.) (Bitplane, Zurich, Switzerland).

### 2.8. Mice

For in vivo experiments, eight- to ten-week old female BALB/c mice (BALB/cAnNCrl) were used. The animals were obtained from Charles River Laboratories (Charles River, Calco (Lecco), Italy). All procedures were conducted in accordance with the European Union (EU) directive (2010/63/EU) and executed according to the 3R principle and the ARRIVE∙b ×∙c∙π/6. When the tumors reached a volume of approximately 80 mm^3^, mice were randomly assigned into one of two groups: a control (non-irradiated) group (*n* = 13) and an irradiated (treated) group (*n* = 16), which received a fractionated IR dose of 5 × 5 Gy, consistent with clinically relevant regimens. Mice were sacrificed at a predetermined timepoint (*n* = 3 per timepoint) or when the tumor volume reached 500 mm^3^ (*n* = 10). Additionally, body weight and mice behavior were assessed via the mouse grimace scale as an indicator of the systemic toxicity of the RT. Tumor response to IR was analyzed in R, using the DRAP package [34].

### 2.9. Tumor Collection

Tumors were collected at 24 and 48 h following the final dose of 5 × 5 Gy IR. Mice were euthanized, and tumors were surgically excised. The excised tumors were fixed in 2% paraformaldehyde (Thermo Fisher) for 16 h at 4 °C, followed by incubation in 30% sucrose solution for 24 h at 4 °C. Subsequently, tumors were embedded in optimal cutting temperature (OCT) compound, snap-frozen in dry ice, and stored at −80 °C until further analysis.

### 2.10. Immunofluorescence In Vivo

Frozen tumors embedded in OCT compound were sectioned at a thickness of 10 µm using a Cryostat 1850 (Leica, Wetzlar, Germany) and immunofluorescently stained to assess tumor endothelial cell (TEC) activation and immune response markers. All staining steps were conducted at room temperature unless otherwise specified, with each wash step performed in 1× PBS for 5 min. Sections were first dried for 10 min at 37 °C and washed twice. Antigen retrieval was performed in a pre-warmed (95 °C) sodium citrate buffer (10 mM sodium citrate, 0.05% Tween 20, pH 6.0) for 30 min, followed by cooling at room temperature. Tissue sections were washed once and permeabilized in blocking buffer 1 (0.5% Tween 20, 5% donkey serum, 22.52 mg/mL glycine in PBS) in a humidified chamber for 30 min. Next, the sections were incubated for 1 h in blocking buffer 2 (5% donkey serum, 22.52 mg/mL glycine in PBS) in a humidified chamber, followed by overnight incubation in primary antibodies. Prior to antibody incubation, primary antibodies for NF-κβ (Abcam, #ab140751), STING (Novus Biologicals, #NBP2-24683), and CD31 (R&D Systems, #AF3628) were diluted in blocking buffer 2, at a dilution of 1:200. The next day, samples were washed three times and incubated with secondary antibodies Alexa Fluor 488 (Jackson ImmunoResearch, #711-545-155 and #703-545-155), Alexa Fluor 647 (Jackson ImmunoResearch, #127-605-099), and Alexa Fluor Cy3 (Jackson ImmunoResearch #712-165-152 and 711-165-153), diluted to 1:400 in 1X PBS, for 1h in a dark humidified chamber. After three additional washes, nuclei were counterstained with Hoechst 33342 solution (3 mg/mL) (Thermo Fisher) in 1X PBS for 10 min in a dark humidified chamber, followed by a final series of three washes. Tumor sections were mounted using ProLong Glass Antifade Mountant (Thermo Fisher) and imaged using an LSM 800 confocal microscope (Carl Zeiss, Oberkochen, Germany) equipped with a 63× oil immersion objective (NA 1.4), utilizing image acquisition settings as described in Section 2.7. Three or more randomly selected fields of view were imaged per tumor section, and three independent tumors were analyzed per treatment group (*n* = 3 per group). Image visualization and analysis were performed using Imaris software (v9.9.1) (Bitplane, Belfast, UK).

### 2.11. Statistics and Reproducibility

Statistical analysis of the data was performed using GraphPad Prism 10 software (GraphPad Software). Values were expressed as arithmetic mean (AM) ± standard error of the mean (SEM). The normality of each dataset was calculated using the Shapiro–Wilk test, which is appropriate for small datasets. For cell proliferation and cell survival experiments, statistical significance between the control and treatment groups was calculated using the unpaired parametric *t*-test for normally distributed data and the non-parametric Mann–Whitney test for non-normally distributed data. For comparisons involving multiple groups, the multiple parametric *t*-test was used for normally distributed data and non-parametric *t*-test for non-normally distributed data. *p*-values below 0.05 were considered statistically significant. The number of biological replicates per group in all experiments was ≥3.

## 3. Results

### 3.1. IR Reduces the Proliferation and Survival of Murine EC Lines

**Figure 1:**
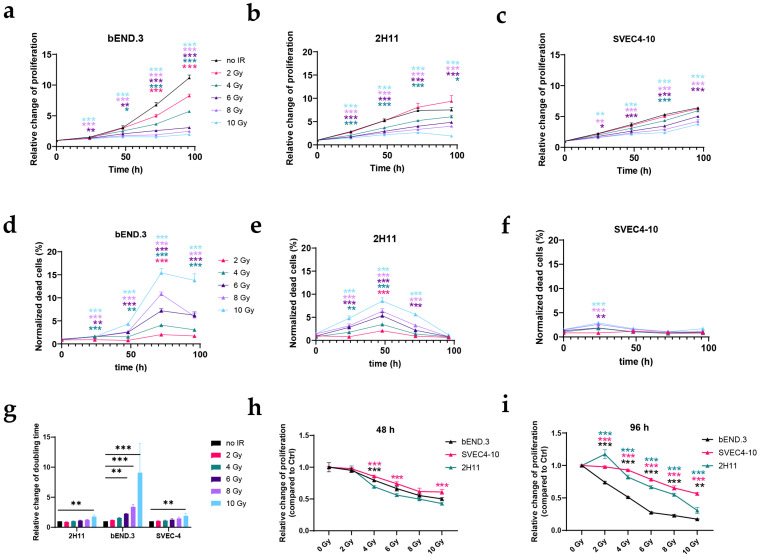
Murine EC proliferation and survival after 2–10 Gy of IR. Murine EC lines were irradiated with doses from 2 to 10 Gy. The number of Hoechst and PI-positive nuclei was measured on the Cytation 1 Cell Imaging Multi-Mode Reader at 0, 24, 48, 72, and 96 h after IR. (**a**–**c**) Relative change in proliferation in comparison with t = 0 h for bEND.3 (**a**), 2H11 (**b**), and SVEC4-10 (**c**). (**d**–**f**) Percentage of dead cells at each timepoint, compared with control (no IR) samples for bEND.3 (**d**), 2H11 (**e**), and SVEC4-10 (**f**). (**g**) Relative change in doubling time for each IR dose, in comparison with control (no IR) samples. (**h**,**i**) Relative change in proliferation (compared with control samples) at 48 h (**h**) and 96 h (**i**) after IR, used to compare radiosensitivity between each of the murine EC lines tested. *p*-values were calculated using a multiple unpaired *t*-test with a false discovery rate (FDR) (**a**–**f**,**h**,**i**) and an ordinary one-way ANOVA, followed by Dunnett test for multiple comparisons (**g**). Statistical significance was considered as *p* < 0.05 (*), *p* < 0.01 (**), and *p* < 0.001 (***). In (**h**,**i**), statistical significances between each EC line is shown in colors: 2H11 vs. bEND.3 in black, 2H11 vs. SVEC4-10 in pink, and SVEC4-10 vs. bEND.3 in green. Error bars represent ± standard error (SEM). *n* = 3 per group. EC—endothelial cell. IR—irradiation.

In order to determine the effect of IR on the proliferation and survival of ECs in vitro, we irradiated murine bEnd.3, 2H11, and SVEC4-10, and human EA.hy926, Hulec5a, and HUVEC EC lines with single doses from 2 to 10 Gy and followed their growth and cell death kinetics using daily imaging of cells stained with Hoechst and propidium iodide to differentiate between live and dead cells (Supplementary Figure S1). Among the murine EC lines, bEnd.3 was the most radiosensitive, followed by 2H11 and SVEC4-10 (Figure 1). In the bEnd.3 EC line, a reduced proliferation was already determined after the 2 Gy single-dose IR (Figure 1a); whereas, in the 2H11 and SVCE4-10 EC lines reduced proliferation was determined after 4 Gy single-dose IR (Figure 1b,c). In all three EC lines, the effect of IR on proliferation was dose-dependent, with higher doses having a more profound effect, which was also statistically significant at earlier timepoints. Similarly, the percent of dead cells confirmed that the most radiosensitive EC line was bEnd.3, as the percentage of dead cells was statistically significantly increased in all timepoints after IR with 4–10 Gy, which was not the case in 2H11 and SVEC4-10 (Figure 1d–f). In all EC lines, the most pronounced effect was observed after IR with 10 Gy, with the peak incidence in cell death occurring 72, 48, or 24 h after exposure in bEnd.3, 2H11, and SVEC4-10, respectively, followed by a decline in PI-positive cells (Figure 1d–f). This suggests that the rate of IR-induced cell death is not only dose- and time-dependent, but also cell line-dependent. In control samples, the doubling times were 26.91 ± 1.44 h for bEND.3, 39.93 ± 6.75 h for 2H11, and 37.91 ± 5.90 h for SVEC4-10. In all murine EC lines tested, the doubling time was significantly prolonged after IR with 10 Gy; however, in bEnd.3 cells, a significantly longer doubling time was also observed after IR with 6 and 8 Gy (Figure 1g). To directly compare the radiosensitivity of these EC lines, we calculated the relative change in proliferation for each dose at 48 h and 96 h after IR as an indicator of the radiosensitivity of the cells (Figure 1h,i). At 48 h after IR, there was no significant difference between radiosensitivity in bEnd.3 and SVEC4-10, regardless of the IR dose. At the same timepoint, radiosensitivity of the 2H11 cell line was significantly different from the SVEC4-10 cell line, in the case of IR with 4-6 Gy (Figure 1h). On the other hand, regardless of the IR dose, 96 h after IR, the radiosensitivity of bEnd.3, SVEC4-10, and 2H11 were significantly different among each other (Figure 1i), confirming that the most radiosensitive murine EC line was bEnd.3, followed by 2H11, and SVEC4-10 being the least radiosensitive one.

### 3.2. IR Reduces the Proliferation and Survival of Human EC Lines

**Figure 2:**
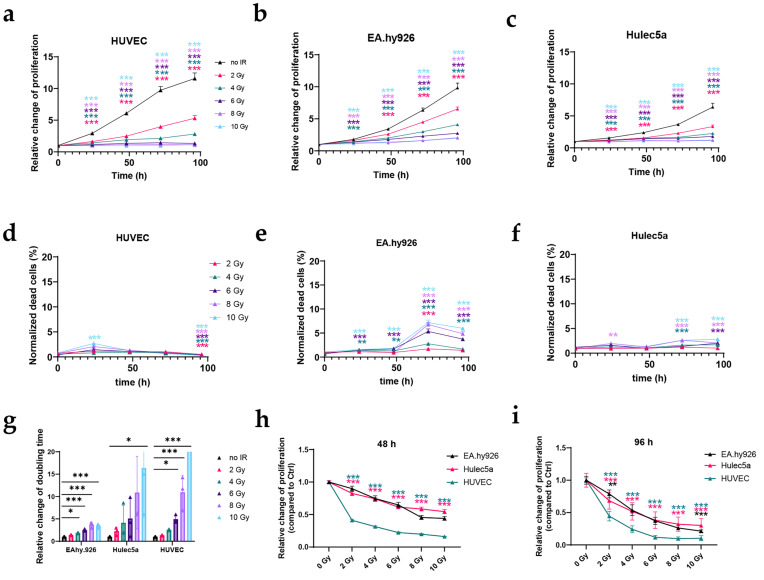
Proliferation and survival of human EC following 2–10 Gy of IR. Human EC lines were exposed to IR doses from 2 to 10 Gy. Hoechst- and PI-positive nuclei were quantified using the Cytation 1 Cell Imaging Multi-Mode Reader at 0, 24, 48, 72, and 96 h after IR. (**a**–**c**) Relative changes in proliferation compared with the starting point (t = 0 h) are shown for HUVECs (**a**), EA.hy926 (**b**), and Hulec5a (**c**). (**d**,**f**) The percentage of dead cells at each timepoint relative to the control (no IR) samples for HUVECs (**d**), EA.hy926 (**e**), and Hulec5a (**f**). (**g**) Relative changes in doubling time for each IR dose, compared with the control (no IR) samples. (**h**,**i**) Relative proliferation changes at 48 h (**h**) and 96 h (**i**) after IR, showing differences in radiosensitivity among the tested human EC lines. Statistical analysis was performed using multiple unpaired *t*-tests with FDR adjustment for (**a**–**f**) and (**h**,**i**), and ordinary one-way ANOVA with Dunnett’s test for multiple comparisons for (**g**). Statistical significance was defined as *p* < 0.05 (*), *p* < 0.01 (**), and *p* < 0.001 (***). In (**h**,**i**), statistical significances between each EC line are shown in colors: EA.hy926 vs. Hulec5a in black, Hulec5a vs. HUVECs in pink, EA.hy926 vs. HUVECs in green. Error bars indicate ± standard error (SEM). *n* = 3 per group.

Among all tested human EC lines, the HUVEC line was the most radiosensitive, while the radiosensitivity of EA.hy926 and Hulec5a was comparable (Figure 2). A statistically significant decrease in proliferation, compared with the control, was observed regardless of the dose and timepoint, with the exception of EA.hy926 at 24 h after IR with 2 Gy (Figure 2a–c). In line with the results from murine EC lines, the effect of IR on cell death kinetics was time-, dose-, and cell line-dependent. The most pronounced effect on cell death was determined in EA.hy926, 72 h after IR with 2–10 Gy, followed by HUVECs (24 h after IR), and Hulec5a (96 h after IR). In EA.hy926 cells, a statistically significant increase in the percent of dead cells after IR with 4, 6, and 10 Gy was also observed at earlier timepoints, which was not the case in HUVECs and Hulec5a cells (Figure 2d–f). Doubling times calculated for the control ECs were 28.17 ± 2.44 h for HUVECs, 25.31 ± 1.57 h for EA.hy926, and 31.87 ± 4.39 h for Hulec5a. IR with 10 Gy caused significantly prolonged doubling times in all tested human EC lines, while a significantly prolonged doubling time after IR with lower doses was determined in EAhy.926 (4–8 Gy), and HUVEC (6 and 8 Gy) cell lines (Figure 2g). A direct comparison of relative change in proliferation between the EC lines, as an indicator of the radiosensitivity of the cells, showed that HUVECs were the most radiosensitive EC line as their relative proliferation was significantly reduced compared with both EA.hy926 and Hulec5a cell lines after IR with 2–10 Gy at 48 h and 96 h after IR (Figure 2h–i). In contrast, there was almost no difference in the radiosensitivity between the EA.hy926 and Hulec5a cell lines, as it was statistically different only at 96 h after 12 and 10 Gy of IR (Figure 2h–i).

### 3.3. IR Changes the Global Transcriptomic Profile of HUVEC Line

To understand the dependence of HUVEC gene expression on IR dose and time after IR, we compared the RNA transcriptomic profiles of HUVECs exposed to either 2 or 5 Gy with corresponding control (non-irradiated) samples. RNA samples were collected and analyzed either 24 h or 72 h after IR. Additionally, we assessed the temporal dynamics of HUVEC gene expression by comparing the transcriptomic profiles of HUVECs at 72 h versus 24 h after IR at the same doses.

**Figure 3:**
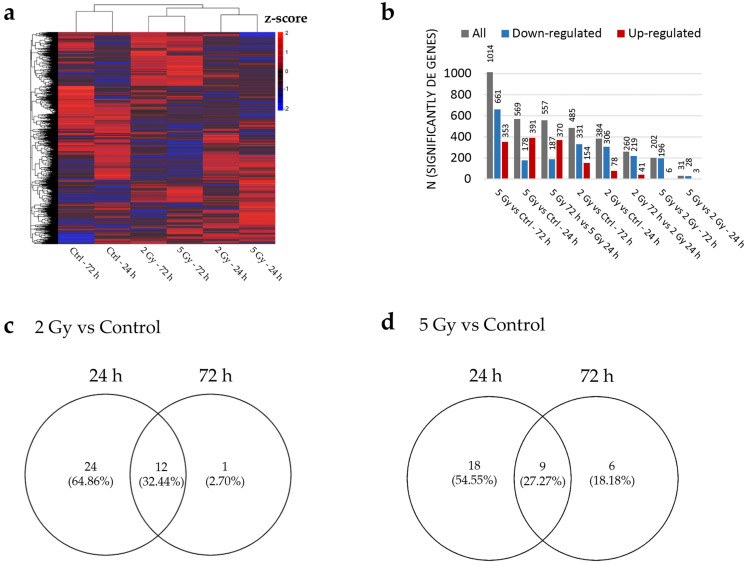
Dose- and time- dependency of the HUVEC transcriptomes on IR. HUVECs were exposed to 2 or 5 Gy IR, and RNA was isolated and sequenced at 24 or 72 h after IR. The global transcriptomic profiles of irradiated samples were compared with their corresponding controls (no IR) and across experimental conditions (dose and time after the exposure). (**a**) Heatmap presenting unsupervised hierarchical clustering of all genes across control and irradiated samples. Gene expression values were normalized and scaled to Z-scores to highlight relative expression changes. (**b**) Bar plot presenting the number of significantly differentially expressed genes in each comparison. Adjusted *p*-value of <0.05 and fold change (FC) of >1.5 (up-regulation) or FC of <0.67 (down-regulation) were considered as significance thresholds. (**c**,**d**) Venn diagrams presenting number of significantly enriched pathways identified by KEGG analysis shared between samples analyzed at 24 and 72 h after 2 Gy (**c**) and 5 Gy (**d**) (See Supplementary Tables S1 and S2). Enrichment was considered statistically significant at *p*-values of <0.05. *n* = 5 per group. KEGG—Functional Kyoto Encyclopedia of Genes and Genomes.

PCA revealed no clear clustering between samples irradiated with 5 or 2 Gy at either of the analyzed timepoints (Supplementary Figure S2a,b). However, within the same dose, the transcriptomic profiles of samples analyzed 24 h after IR clustered apart from those analyzed 72 h after IR (Supplementary Figure S2c,d). This suggests that the HUVEC transcriptomic profile after IR is more time- than dose-dependent, which is also presented on a heatmap showing normalized counts of all genes after filtering (Figure 3a). DGE analysis revealed a more pronounced effect of IR on HUVEC global gene expression at a dose of 5 Gy compared with 2 Gy, resulting in 1014 (661 down-regulated and 353 up-regulated) significantly differentially expressed (DE) genes. However, even a lower dose of 2 Gy of IR affected global gene expression, with 569 (178 down-regulated and 370 up-regulated) DE genes (Figure 3b). Furthermore, regardless of the dose, a greater effect of IR was observed at 72 h compared with 24 h after IR (Figure 3b). The comparative analysis of samples irradiated with 5 Gy versus 2 Gy revealed a small number of DE genes at both timepoints (31 DE genes at 24 h and 202 genes at 72 h timepoint) (Figure 3b); therefore, these comparisons were excluded from further analysis.

To elucidate the contribution of IR on the regulation of signaling pathways, KEGG analysis was performed [30,31]. Irrespective of the dose and time after IR, the majority of the top 20 significantly down-regulated pathways observed in irradiated samples were associated with cell cycle regulation, including nucleotide excision repair, mismatch repair, and DNA replication (Supplementary Figure S3a–d). Conversely, among the top 20 significantly up-regulated pathways, the immune response-related pathways, such as cytokine–cytokine receptor interaction, TNF signaling, and NF-κβ signaling pathways, were present (Supplementary Figure S3e–h). To delineate the temporal dynamics in the usage of signaling pathways, the significantly enriched pathways of samples irradiated with the same dose but whose transcriptomes were analyzed at different timepoints were compared (Figure 3c,d, Supplementary Tables S1 and S2). Regardless of the dose, some of the pathways, such as the significantly up-regulated p53 signaling pathway and significantly down-regulated cell cycle and DNA replication, were enriched at both the 24 h and 72 h timepoints (relative to the control), suggesting their importance in evolving response to IR (Figure 3c,d, Supplementary Tables S1 and S2). However, several pathways were specifically enriched only at either 24 h or 72 h after IR, including significantly up-regulated NF-κβ signaling and hematopoietic cell lineage and significantly down-regulated nucleocytoplasmic transport at 24 h after IR and up-regulated lysosome pathway at 72 h after IR with 2 or 5 Gy. These results indicate that distinct pathways are activated at different times after IR (Figure 3c,d, Supplementary Tables S1 and S2).

### 3.4. IR Modifies the Cell Cycle-Related Transcriptomic Profile of HUVEC Line

**Figure 4:**
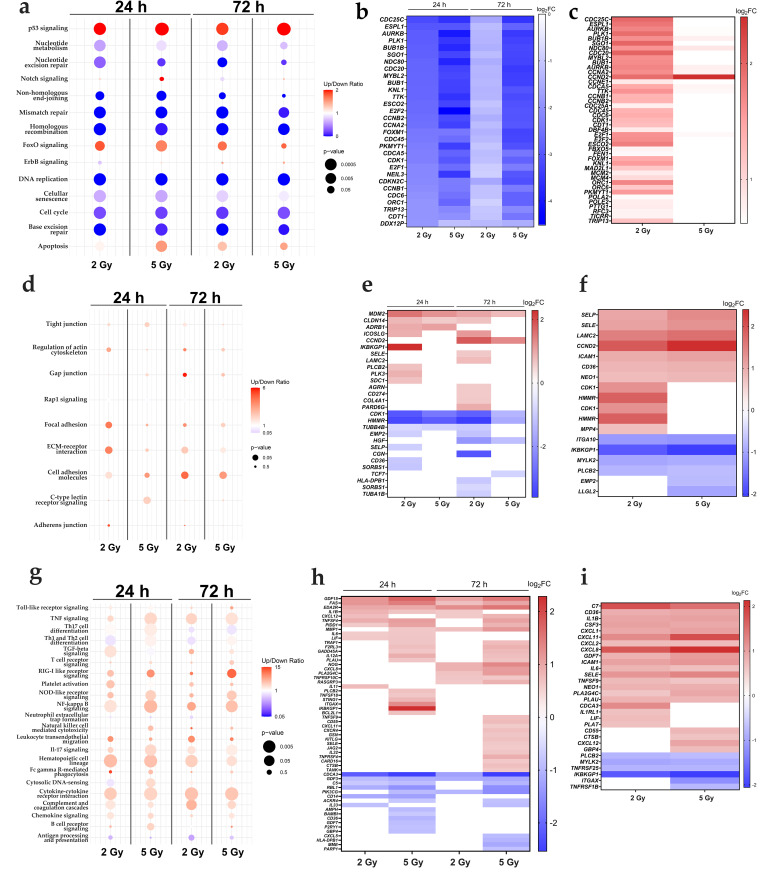
Transcriptomic profiles of HUVECs associated with EC activation after 2 Gy and 5 Gy of IR. Transcriptomic changes in irradiated HUVECs, analyzed in comparison with their respective controls (no IR) and across experimental conditions (varying dose and time after IR). Specific transcriptomic profiles related to cell cycle (**a**–**c**), ECM remodeling (**d**–**f**), and immune response activation (**g**–**i**). (**a**,**d**,**g**) Dot plots displaying pathways enriched in irradiated HUVECs compared with the control, identified through KEGG pathway analysis. Up/Down Ratio represents a proportion between up- and down-regulated genes in each pathway. Up-regulated pathways are defined with Up/Down ratio >1, and down-regulated with Up/Down ratio <1. Pathways with *p*-values of <0.05 were considered as statistically significant. (**b**) Top 30 significantly cell cycle-associated differentially expressed genes in irradiated samples compared with the control. (**c**,**f**,**i**) Heatmaps illustrating significantly differentially expressed genes related to the cell cycle (**c**), ECM (**f**), and immune response (**i**) at 72 vs. 24 h after IR. (**e**,**h**) Heatmaps of significantly differentially expressed genes in irradiated samples relative to the control, related to ECM (**e**) and immune response (**h**). Genes were considered significantly differentially expressed if the adjusted *p*-value was <0.05 and fold change (FC) was >1.5 (up-regulation) or FC was <0.67 (down-regulation). *n* = 5 per group. IR—irradiation. EC—endothelial cell. KEGG—Functional Kyoto Encyclopedia of Genes and Genomes.

To further investigate the effects of IR on EC proliferation, the IR-induced changes in the cell cycle-related transcriptome of HUVECs was evaluated. Irrespective of the dose or time after IR, the cell cycle progression-related signaling pathways such as DNA replication, base excision repair and cell cycle were significantly down-regulated (Figure 4a). Contrary to that, the signaling pathways related to cell cycle arrest were up-regulated after IR, with p53 signaling being up-regulated regardless of the dose of IR and time after IR, whereas the FoxO signaling pathway was up-regulated only after 2 Gy of IR (Figure 4a). Further, all of the top 30 significant DE genes related to the cell cycle were down-regulated in the irradiated samples (Figure 4b, Supplementary Table S3). These genes were involved in each of the cell cycle phases, including *E2F1* and *CDKN2C* (G1 phase), *CDC6* and *CDT1* (S phase), *CCNA2* and *BUB1* (G2 phase), and *AURKB* and *BUB1B* (M phase) (Figure 4b, Supplementary Table S3). Interestingly, *CDKN1A*, a gene encoding for p21, was significantly up-regulated regardless of the dose and time after IR (Supplementary Table S4). A comparison of cell cycle-related genes between 72 h and 24 h after 2 Gy of IR revealed 43 significantly up-regulated genes and no significantly down-regulated genes (Figure 4c, Supplementary Table S4). Interestingly, we observed only 10 significantly up-regulated genes and no significantly down-regulated genes 72 h after IR with 5 Gy compared with 24 h after IR with the same dose (Figure 4c, Supplementary Table S4). These results suggest that the effect on cell cycle transcriptome of HUVECs is more pronounced at 72 h than 24 h after IR with 2 Gy; however, IR with 5 Gy prolongs these changes up to 72 h after IR (Figure 4c, Supplementary Table S4).

### 3.5. IR Changes the ECM-Related Transcriptomic Profile of HUVEC EC Line

To access whether IR alone could lead to EC activation, the genes and pathways associated with activated endothelium, including pathways related to changes in the ECM were interrogated. Compared with the control group, a significant up-regulation of cell adhesion molecules, ECM–receptor interaction, focal adhesion pathways, and the regulation of actin cytoskeleton were observed 24 h after IR with 2 Gy (Figure 4d). However, 72 h after 2 Gy of IR, only cell adhesion and ECM–receptor interaction pathways remained significantly up-regulated (Figure 4d). Contrary to that, IR with 5 Gy did not induce a similarly pronounced effect on ECM-related pathways in HUVECs, with only the C-type lectin receptor signaling pathway being up-regulated at 24 h and the cell adhesion molecule pathway at 72 h after IR (Figure 4d). Despite KEGG analysis showing that 2 Gy of IR has a more pronounced effect on the ECM-associated transcriptome, the DGE analysis of ECM-related genes revealed a higher number of significantly changed genes after IR with 5 Gy at both observed timepoints. At 72 h after IR with 5 Gy, up-regulation of genes involved in cell adhesion, such as *SELE* (log_2_FC = 0.62), *LAMC2* (log_2_FC = 0.76), and *PARD6G* (log_2_FC = 0.94), as well as genes supporting cell migration, such as *MDM2* (log_2_FC = 1.01), were determined (Figure 4e, Supplementary Table S5), whereas the genes associated with cytoskeletal organization and intracellular trafficking, such as *TUBB4B* (log_2_FC = −0.75) and *TUBA1B* (log_2_FC = −0.80), were significantly down-regulated (Figure 4e, Supplementary Table S5). Notably, a significant effect of 5 Gy of IR on the ECM-related transcriptome in HUVECs was also observed 24 h after IR, with *EMP2* (log_2_FC = −0.73), *SDC1* (log_2_FC = 0.70), and *SORBS1* (log_2_FC = −0.90) being significantly changed only at this timepoint, highlighting their importance in the early response to IR (Figure 4e, Supplementary Table S5). Finally, we compared the expression of ECM-related pathways between 72 h and 24 h after IR for both tested doses (Figure 4f, Supplementary Table S6). Compared with 24 h, 72 h after IR with either 2 Gy or 5 Gy, genes associated with cell adhesion and consequent leukocyte rolling, such as *SELP* (log_2_FC = 1.06 and log_2_FC = 1.37 after 2 or 5 Gy of IR, respectively), *SELE* (log_2_FC = 1.10 and log_2_FC = 1.29), *LAMC2* (log_2_FC = 1.50 and log_2_FC = 1.69), and *ICAM-1* (log_2_FC = 0.98 and log_2_FC = 1.09), were significantly up-regulated (Figure 4f, Supplementary Table S6). Contrary to that, *ITGA10* (log_2_FC = −0.94 and log_2_FC = −1.00), *MYLK2* (log_2_FC = −0.82 and log_2_FC = −0.79), and *PLCB2* (log_2_FC = −0.66 and log_2_FC = −0.60), involved in cell–ECM interactions, were significantly down-regulated at 72 h (Figure 4f, Supplementary Table S6). Altogether, these results indicate that IR caused dose- and time-dependent changes associated with ECM in HUVECs, in a manner supportive to cell adhesion and immune cell rolling.

### 3.6. IR Up-Regulates the Immune Response-Related Transcriptomic Profile of HUVEC EC Line

To access whether IR alone could lead to the changes in ECs favorable for the activation of the immune system, the genes and pathways specific for the activation of the immune response were interrogated [9,10]. IR of HUVECs activated pathways related to the immune response at the transcriptional level. Regardless of the dose, significant up-regulation of the innate immune response, including NF-κβ signaling, TNFα signaling, and cytokine–cytokine receptor interaction pathways, was observed at 24 h after IR. At the same timepoint, independently of the dose, a significant up-regulation of pathways associated with the adaptive immune response, such as Th1, Th2, and Th17 cell differentiation, was determined. This suggests that adaptive immune response and pro-inflammatory pathways are activated in irradiated ECs as early as 24 h after IR (Figure 4g). Interestingly, while pathways associated with the innate immune response remained significantly up-regulated at 72 h after IR, there was no significant up-regulation of adaptive immune response pathways at this later timepoint, regardless of the dose (Figure 4g). The expression of genes related to the innate immune response was dose-dependent. While both doses led to significant up-regulation of interleukins (*IL-1B*, *IL-6*) 24 h after IR, only 5 Gy additionally caused up-regulation of tumor necrosis factors, such as *TNFSF18* (log_2_FC = 0.65) and *TRAF1* (log_2_FC = 0.65) and *STING1* (log_2_FC = 0.81), one of the main actors in DNA sensing (Figure 4h, Supplementary Table S7). At 72 h after IR with 5 Gy, there was an up-regulation of chemokines *CXCL11* (log_2_FC = 0.60), *CXCL12* (log_2_FC = 0.845), *CXCR4* (log_2_FC = 0.72), and *CXCL8* (log_2_FC = 1.10) and tumor necrosis factors, such as TNFSF4 (log_2_FC = 1.10) (Figure 4h, Supplementary Table S7). To assess how the IR-induced activation of immune response-related pathways progresses over time, the expression of immune-related genes between samples analyzed at 72 h versus 24 h after IR was compared. Chemokines such as *CXCL1* (log_2_FC = 0.92 and log_2_FC = 1.00 after 2 or 5 Gy of IR, respectively), *CXCL2* (log_2_FC = 0.72 and log_2_FC = 0.73), and interleukins *IL-1B* (log_2_FC = 0.96 and log_2_FC = 0.99), *IL-6* (log_2_FC = 0.80 and log_2_FC = 0.68) were significantly up-regulated at 72 h compared with 24 h after IR with 2 or 5 Gy (Figure 4i, Supplementary Table S8). Finally, qRT-PCR was used to confirm the up-regulation of three selected genes supporting IR-induced HUVEC activation. Similarly to RNA sequencing, *SELE*, *TNFSF4*, and *CXCL12* were up-regulated (Supplementary Figure S4), with *SELE* statically significantly up-regulated at 72 h after IR with 2 Gy (Supplementary Figure S4a), *TNFSF4* at both timepoints and after both IR doses (Supplementary Figure S4b), and *CXCL12* at 48 h after IR with both doses (Supplementary Figure S4c). Interestingly, qRT-PCR showed that *SELE* was down-regulated 24 h after IR with 5 Gy, while the RNA sequencing data showed no statistically significant change in *SELE* expression at this timepoint, suggesting that there may be transient down-regulation that was not detected by the RNA sequencing approach.

### 3.7. IR Modifies the Expression of EC Activation and Immune Response-Related Markers on a Protein Level in HUVEC Line

**Figure 5:**
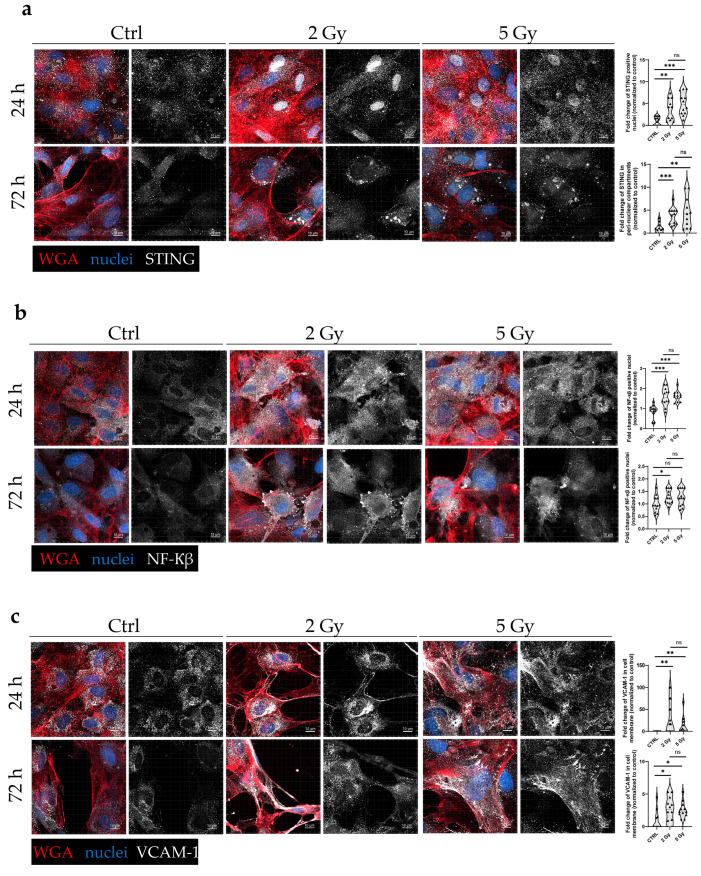
The impact of 2 Gy and 5 Gy IR on proteins associated with EC activation. Representative immunofluorescence images of control and irradiated HUVECs stained for STING (**a**), NF-κβ (**b**), and VCAM-1 (**c**). HUVECs were immunofluorescently stained 24 h or 72 h after IR with either 2 or 5 Gy. Corresponding control samples were stained at the same timepoints (24 h and 72 h). Violin plots accompanying the images represent the quantification of activated STING (**a**), NF-κβ (**b**), and VCAM-1 (**c**). Hoechst 33342 was used to stain nuclei and WGA to stain cell membranes. Activation was calculated as the ratio of the number of cells displaying translocation of the target protein to the total number of cells per image, with values normalized to control. Statistical significance was determined using the unpaired *t*-test for normally distributed data and the Mann–Whitney test for not normally distributed data. Significance is indicated as *p* < 0.05 (*), *p* < 0.01 (**), and *p* < 0.001 (***). Scale bar: 10 µm. *n* = 3 per group. STING—Stimulator of Interferon Response CGAMP Interactor 1. NF-κβ—nuclear factor kappa β. VCAM-1—vascular cell adhesion molecule 1. WGA—wheat germ agglutinin. ns—not significant.

To determine how the dose of IR affects EC activation, immunofluorescence staining of several proteins involved in this process in HUVECs was performed at different times after IR. To understand the interconnectivity between IR-induced DNA sensing, its downstream pathways, and changes in ECM, the irradiated HUVECs were stained for STING, NF-κB, and VCAM-1 proteins. The activation of STING is primarily characterized by its translocation to peri-nuclear punctate structures [9,10,35], which were observed in irradiated (2 and 5 Gy) HUVECs, 72 h after IR. In contrast, in the control, non-irradiated HUVECs, STING staining was dispersed throughout the cytoplasm (Figure 5a); 72 h after 2 Gy of IR, the proportion of peri-nuclear STING increased by 3.73 ± 0.52-fold, and after 5 Gy of IR, it increased by 4.49 ± 1.00-fold compared with the control (Figure 5a). Interestingly, at 24 h after IR, the translocation of STING from the cytoplasm into the nucleus was observed, indicated by the increase in the number of STING-positive nuclei by 4.32 ± 0.99-fold (2 Gy) and 4.95 ± 0.73-fold (5 Gy) in comparison in the control group (Figure 5a). Further, the expression levels of NF-κβ, a crucial downstream transcriptional factor of STING signaling, whose activation is characterized by protein translocation from the cytoplasm to the nucleus was then determined. There was a statistically significant difference in the number of NF-κβ positive nuclei at the 24 h timepoint (compared to the control), regardless of the IR dose (Figure 5b). However, at 72 h after IR there was a significant increase in nuclear accumulation of NF-κβ only after 2 Gy of IR, with a 1.28 ± 0.08-fold increase compared with the control group (Figure 5b). NF-κβ signaling facilitates the expression of adhesion molecules, such as VCAM-1, the activation of which is characterized by its translocation to the plasma membrane [21,36]. This was also the case in HUVECs after IR, as the increase in cells with VCAM-1 present in the plasma membrane was statistically significant at 24 h after IR with 2 Gy of IR, demonstrating a more pronounced effect than 5 Gy of IR, with 29.90 ± 9.04-fold and 13.00 ± 6.14-fold increases comparing with the control group, respectively (Figure 5c). An increase in the number of cells with VCAM-1 present in the plasma membrane at 72 h after IR was less pronounced than after 24 h (Figure 5c).

### 3.8. IR Affects STING Signaling-Related Transcriptomic Profile of HUVEC Line

**Figure 6:**
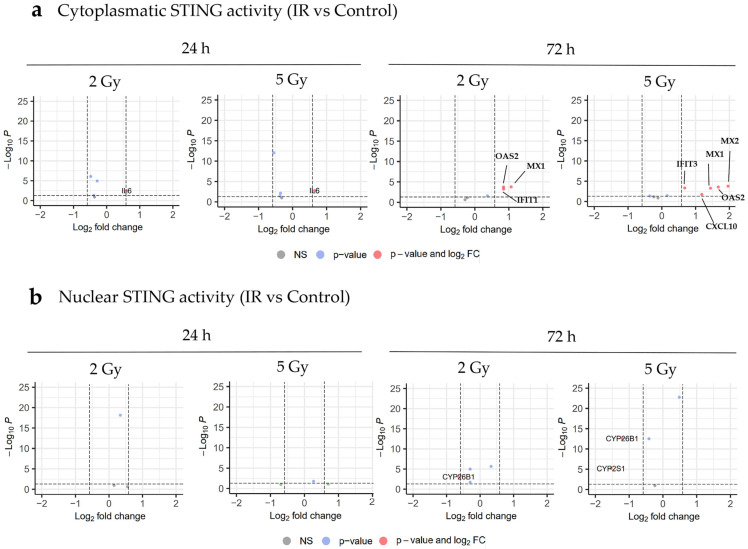
STING-related RNA expression profiles in HUVECs irradiated with 2 Gy or 5 Gy. (**a**,**b**) Volcano plots presenting differentially expressed genes associated with cytoplasmic (**a**) and nuclear (**b**) STING signaling in samples irradiated with 2 Gy or 5 Gy, compared with the control (no IR) samples. Genes with adjusted *p*-values of <0.05 and a fold change (FC) of >1.5 (up-regulation) or FC of <0.67 (down-regulation) were considered as significantly differentially expressed (red—up-regulated genes, blue—down-regulated genes, gray—not significant). Genes not presented on the plots were filtered out due to low expression levels across all samples. All STING-associated genes analyzed are listed in *n* = 5 per group. NS—not significant. FC—fold change. IR—irradiation.

To further explore our findings suggesting that IR induces the translocation of STING into the nuclei (24 h after IR) or to peri-nuclear compartments and cytosol (72 h after IR) (Figure 5a), the genes and pathways associated with STING signaling were further explored. There were no significant changes in the expression of genes regulated by cGAS-STING signaling (excluding *IL-6*) at 24 h after IR with 2 or 5 Gy. However, a significant up-regulation of cGAS-STING signaling downstream genes such as *IFIT1* (log_2_FC = 1.67), *MX1* (log_2_FC = 1.96), *MX2* (log_2_FC = 2.08), and *OAS2* (log_2_FC = 1.44), was determined 72 h after 5 Gy of IR (Figure 6a). Similarly, there was an up-regulation of cGAS-STING signaling-related genes after IR with 2 Gy at the same timepoint, but the effect was less pronounced in comparison to 5 Gy (Figure 6a). Currently, little is known about nuclear STING; however, recent studies have implied that STING forms a complex with the PML Nuclear Body Scaffold (PML) and aryl hydrocarbon receptor (AHR), which occurs in the nucleus and is believed to mainly regulate metabolic processes [35]. Our RNA sequencing analysis did not show significant changes in the regulation of genes potentially associated with STING signaling through PML and AHR, irrespective of the dose, 24 h after IR. However, a down-regulation of AHR signaling downstream genes, such as *CYP26B1* (log_2_FC = −0.64 after 2 Gy and log_2_FC = −1.20 after 5 Gy of IR) with 2 and 5 Gy and *CYP2S1* (log_2_FC = −1.54) after 5 Gy, was determined 72 h after IR (Figure 6b).

### 3.9. Analysis of Publicly Available RNA Sequencing Data of TECs FACS Sorted from MC38 Murine Colon Carcinomas Receiving Single IR Dose of 15 Gy Supports the Observations on IR-Induced Activation of HUVECs In Vitro

**Figure 7:**
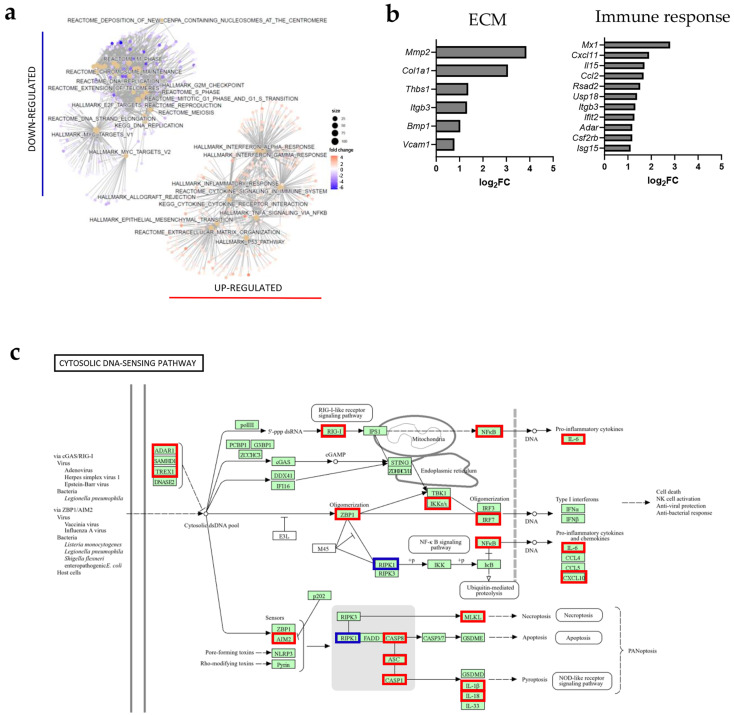
RNA expression profiles of TECs isolated 48 h after IR from MC38 murine colon carcinomas receiving local IR to the tumors while the mice were restrained in (**a**) a lead shield and only the tumors were exposed to the IR. Significantly enriched pathways in irradiated compared with non-irradiated control samples. (**b**) IR-induced significantly up-regulated genes related to changes in ECM and immune response. Genes with adjusted *p*-values of <0.05 and a log_2_FC of >0.5 were considered as significantly up-regulated. (**c**) Significantly differentially expressed genes involved in the cytosolic DNA-sensing pathway (red—up-regulation, blue—down-regulation). *n* = 5 per group. TECs—tumor endothelial cells. FC—fold change. ECM—extracellular matrix. IR—irradiation. See also Supplementary Figure S5 and Table S9.

To understand whether RNA sequencing results obtained from HUVECs are consistent with what is happening in TECs in vivo, we analyzed a publicly available RNA sequencing dataset of TECs isolated 48 h after IR from MC38 murine colon carcinomas receiving a single IR dose of 15 Gy (GSE168481) [7,27]. In line with our in vitro results, IR caused an up-regulation of TEC activation-associated pathways, which were categorized into two groups: ECM change- and immune response-associated pathways. RNA sequencing revealed several up-regulated genes commonly expressed across these pathways, suggesting their involvement in multiple processes during TEC activation (Figure 7a,b, Supplementary Figure S5). To further investigate IR-induced DNA sensing in TECs, which was also observed in vitro, the expression of key DNA sensors was examined. At 48 h after IR, TECs exhibited significant up-regulation of genes involved in DNA sensing and genes directly implicated in STING signaling (Figure 7c, Supplementary Figure S5). Altogether, these findings further support the role of TECs in modulating the IR-induced immune response and suggest the importance of STING signaling in the response of TECs to IR (Figure 7c, Supplementary Figure S5 and Table S9).

### 3.10. IR Induces Activation of STING and NF-κβ in TECs

**Figure 8:**
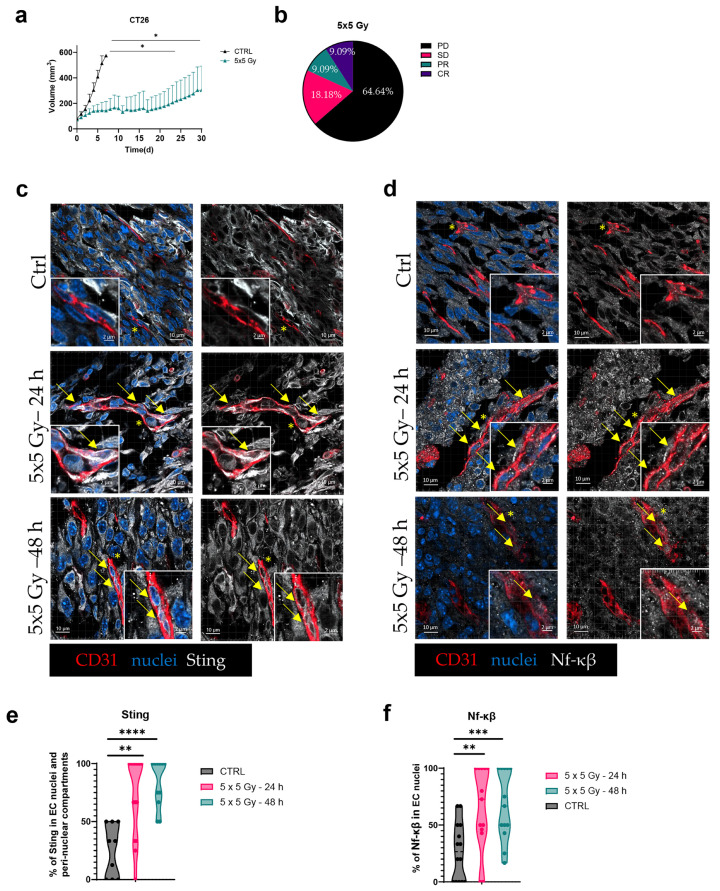
The impact of fractionated 5 × 5 Gy IR on growth of CT26 tumors and activation of STING and Nf-κβ in TECs in vivo. (**a**) Tumor growth following IR with a fractionated dose of 5 × 5 Gy. (**b**) Treatment-response levels of CT26 tumors after IR, based on NPDXE criteria. *n* = 10. PD—progressive disease. SD—stable disease. PR—partial response. CR—complete response. Fractionated 5 × 5 Gy of IR was delivered locally to the subcutaneously growing CT26 tumors while the mice were restrained in a lead shield leaving only the tumors exposed to the IR. Statistical analysis was performed using one-way ANOVA. (**c**–**f**) Representative immunofluorescence images of control and irradiated CT26 tumors at 24 h and 48 h after IR with a fractionated dose of 5 × 5 Gy stained for STING (**c**) and Nf-κβ (**d**). Hoechst 33342 was used to stain nuclei and CD31 as EC markers. Percentage of activated STING (**e**) and Nf-κβ (**f**). Statistical significance was with unpaired *t*-test for normally distributed data. Significance is indicated as: *p* < 0.05 (*) *p* < 0.01 (**), *p* < 0.001 (***), and *p* < 0.0001 (****) compared with the control. Arrows indicate peri-nuclear accumulation of STING (**c**) and nuclear translocation of Nf-κβ (**d**). Asterisks (*) denote magnified regions. Scale bar: 10 µm and 2 µm (magnified insert). *n* = 3 per group.

To determine whether, and at which timepoint, IR induces DNA sensing in TECs and their activation in vivo, the growth of subcutaneously grown CT26 tumors after IR was first evaluated and fresh-frozen sections of irradiated and control (no IR) tumors were stained for STING and Nf-κβ (Figure 8). Firstly, the effects of a fractionated 5 × 5 Gy IR regimen on tumor growth were evaluated in vivo. A significant reduction in tumor volume was observed in irradiated tumors compared with the controls (Figure 8a). The IR responses of mice were assessed using the Novartis Institutes for BioMedical Research PDX encyclopedia (NPDXE) response criteria from the DRAP R package [34]. Although majority of the tumors (64.64%) were identified as progressive disease (PD), IR led to 18.18% of tumors identified as stable disease (SD), and 9.09% of tumors were identified as partial response (PR) and complete response (CR) (Figure 8b). Consistent with our in vitro findings, STING was not expressed in nuclear nor non-nuclear compartments in TECs of the control, non-irradiated tumors. However, following exposure to the 5 × 5 Gy of IR, STING expression was statistically significantly higher throughout the cytoplasm of TECs regardless of the time after IR (Figure 8c,e), with a 21 ± 8.93% and 15 ± 8.99% higher expression at 24 and 48 h, respectively. Furthermore, IR also induced the activation of the downstream STING pathway protein NF-κβ, characterized by its translocation into the nuclei of TECs at both 24 and 48 after IR, with an increase in nuclear Nf-κβ of 24 ± 8.62% (24 h) and 10 ± 8.49% (72 h) (Figure 8d,f).

## 4. Discussion

In this study, we investigated the molecular and functional responses of murine and human endothelial cell lines to IR. We showed that IR doses of 2–10 Gy led to a dose-dependent decrease in proliferation and increased EC death. Transcriptomic analysis of HUVECs irradiated with 2 or 5 Gy showed that the decrease in proliferation was due to down-regulation of cell cycle-related markers and signaling pathways and up-regulation of signaling pathways related to cell cycle arrest. In addition, transcriptome analysis revealed the up-regulation of immune and EC activation markers and signaling pathways, which was also confirmed at the protein level by the detection of higher expressions of EC activation-associated proteins STING, NF-κβ, and VCAM-1 in irradiated HUVECs. Moreover, a similar up-regulation of immune and EC activation markers and signaling pathways was also confirmed in vivo in a publicly available RNA sequencing dataset of murine TECs isolated from irradiated colon carcinoma tumors. Lastly, the increased expression and activation of STING and Nf-κβ was also confirmed in TEC in vivo in irradiated murine colon carcinoma tumors. Taken together, our results suggest that during RT, the irradiated ECs in the TME may contribute to the homing of immune cells into the tumors.

We first investigated the dose- and time-dependent effects of IR doses from 2 to 10 Gy on the proliferation and survival of the murine EC lines bEnd.3, 2H11, and SVEC4-10 and the human EC lines EA.hy926, Hulec5a, and HUVEC. These EC lines differ in their origin, which could explain their different response to IR, as bEND.3 are isolated from the brain vasculature; 2H11 from the bone marrow; and SVEC4-10 from lymph nodes. From the tested human ECs, EA.hy926 are hybrid cells created by fusing HUVECs and cancer cells A549; HUVECs are primary cells isolated from human umbilical veins; whereas Hulec5a are an immortalized cell line derived from human lung microvascular endothelial cells. Interestingly, we did not observe an extensive death of ECs, as the highest observed percent of dead cells was 15% in the bEnd.3 cell line 72 h after 10 Gy of IR. However, a significant decrease in proliferation was observed both in murine and human EC lines. In the murine EC lines, proliferation was reduced at doses of ≥4 Gy, except for the bEnd.3 line where proliferation was significantly reduced already after 2 Gy of IR. A similar effect was observed in the human EC lines; however, the reduction in proliferation was more pronounced compared with the murine EC lines. In agreement with previous studies, our results show that the decrease in EC proliferation correlates with the IR dose [5,17]. Previous research indicated that IR doses of ≥2 Gy induce cell cycle arrest and senescence in ECs, primarily via DNA damage-induced p53 signaling [13,14,15,18,37]. Our transcriptomic analysis confirmed this, showing the up-regulation of p53 signaling in HUVECs 24 and 72 h after IR with 2 and 5 Gy. Interestingly, we also observed an up-regulation of FoxO signaling after 2 Gy of IR, suggesting that in addition to p53, FoxO-regulated proteins such as p21 and p27 may also contribute to IR-induced senescence in ECs. In support of these findings, we observed a down-regulation of DNA replication and cell cycle processes primarily regulated by p21 and p27, as well as an up-regulation of *CDKN1A*, the gene encoding p21, independently of IR dose and timepoint. Thus, the main effect of IR with doses up to 10 Gy on ECs in vitro seems to be the induction of cell cycle arrest leading to senescence and not to the death of the ECs. The relatively long cell cycle arrest observed in our study, as evidenced by a lack of proliferation even 96 h after IR, could also be due to the presence of complex/clustered DNA damage (CDD), defined as two or more DNA lesions within a short distance (typically within one or two helical turns of DNA) on the same or opposite DNA strands. Although CDD is more common after high linear energy transfer (LET) ionizing radiation using particle ions (proton beam therapy, carbon ion therapy, etc.), results of mathematical modeling studies have shown that even after low-LET ionizing radiation, such as X-rays, up to 40% of single-strand breaks (SSBs) and 50% of double-strand breaks (DSBs) may have additional damage nearby and are therefore classified as CDD [38,39]. The slow repair of CDD, which can last up to several days after IR, could, therefore, be one of the reasons for the observed long cell cycle arrest of endothelial cells. However, further studies that accurately quantify the extent and repair dynamics of CDDs in ECs and TECs after IR would be required to provide a definitive answer as to their significance in the response of ECs and TECs to IR. Lastly, the observed activation of NF-κβ could also play an important role in the reduced proliferation of ECs as its activation is generally pro-apoptotic. However, in ECs and TECs, it can exert both pro- and anti-proliferative effects, with the mechanisms underlying this context-dependent behavior still unclear [40,41,42,43]. Therefore, further studies using NF-κβ inhibitors or knock-out cell lines should be performed to elucidate the impact of NF-κβ on EC proliferation after IR.

Our in vitro results on the effects of IR on EC proliferation and cell cycle arrest contribute to the understanding of in vivo responses in normal ECs and TECs. Although previous studies have shown dose- and time-dependent IR effects on TEC in mice, including the apoptosis of ECs in small non-perfused vessels, the ability of surviving ECs to maintain their proliferative capacity has remained unclear [6,7]. Here we show, at the in vitro level, that surviving ECs can maintain their proliferative potential to some extent at IR doses up to 6 Gy, with higher doses severely impairing it. Interestingly, the surviving ECs also show pro-inflammatory properties, suggesting that IR may enhance the anti-tumor immune response by activating endothelial cells. Specifically, we have shown that HUVECs irradiated with 2 or 5 Gy exhibit changes in gene and protein expression characteristic of activated endothelium. Although the exact mechanism of IR-induced endothelial activation is not yet fully understood, it is known that activated ECs release pro-inflammatory chemokines and cytokines that upregulate adhesion molecules and facilitate immune cell recruitment, rolling, and extravasation [13]. The accumulation of cytosolic DNA observed in tumor cells and macrophages after IR may have an important contribution to this process [10]. Activated endothelium is characterized by increased expression of adhesion molecules like VCAM-1, ICAM-1, P-selectin, and E-selectin, partly due to IR-induced NF-κβ activation and high levels of pro-inflammatory cytokines such as TNFα and IL-68. Interestingly, bioinformatic analysis of the gene expression of genes involved in the immune and inflammatory response of cancer and normal tissue to IR revealed that among the 24 marker genes common to the response of normal and tumor tissue to IR, several are associated with endothelial activation, such as *Vcam-1*, *Icam-1*, *E-selectin*, as well as the NF-κβ protein family [44]. Moreover, the IR-induced activation of IκB kinase/inhibitor of NF-κB/NF-κB (IKK/IκB/NF-κB) was recently shown to play a crucial role in the radiation-induced inflammatory response of the spleen, suggesting its importance in the response of various tissues to IR. Whether scavenging ROS after IR would attenuate the effects of IKK/IκB/NF-κB activation in ECs and TECs, as shown in murine spleen models, needs to be further explored. However, it may represent a potentially effective strategy to protect normal blood vessels located in the irradiated area from radiation-induced injury [45]. These results confirm the importance of IR-induced changes in TECs and their activation, as up-regulation of marker genes for activated endothelium can also be detected when analyzing gene expression changes in the whole tumor and not only in TECs. Hallahan DE et al. and Cervelli T et al. demonstrated NF-κβ up-regulation at 6 and 18 h after IR [16,41]. This is consistent with our results, as our transcriptomic analysis of irradiated HUVECs shows an up-regulation of the NF-κβ pathway 24 h after IR with 2 or 5 Gy, which persists 72 h after 5 Gy of IR, suggesting it may play a primary role in the initiation of IR-induced EC activation. We also observed an up-regulation of *STING1* and downstream genes associated with DNA sensing, including *IL-1B*, *IL-6*, *IL-12A*, and *TNFα*, 24 h after 5 Gy of IR, suggesting that IR causes a positive loop of NF-κβ signaling through DNA-damage-activated STING. Additionally, our RNA sequencing analysis of the publicly available dataset on in vivo TECs isolated from murine MC38 colon carcinomas irradiated with a single dose of 15 Gy showed consistent results, including the up-regulation of DNA-sensing pathways and the genes involved, such as *Rig1*, *Irf7*, *Nf-κβ*, and *Il-6*. Furthermore, the single dose of 15 Gy IR delivered only to the subcutaneously growing tumors, while the mice are shielded by a lead restrainer leaving only the tumor exposed to IR, was not lethal to mice, as has already been previously reported, when even single doses higher than 40 Gy were delivered locally to subcutaneously growing tumors; however, dry desquamation of the skin in the irradiated area became significant at doses above 15 Gy [46]. We further confirmed our transcriptomic results at the protein level using immunofluorescence staining of HUVECs and irradiated CT26 murine colon carcinomas. In the case of HUVECs, and regardless of the IR dose and time after IR, STING and NF-κβ were activated. Interestingly, STING translocated to the nuclei at 24 h, but not at 72 h after IR, suggesting that its role in the early response to IR may not be linked to the cytosolic cGAS-STING signaling pathway. Instead, an alternative STING signaling pathway that has not yet been observed in irradiated ECs may play a role. Interestingly, this was also determined in our in vivo results, with STING presence in nuclei of murine TECs, at 24 h after IR, and its absence at 48 h after IR and in non-irradiated samples. Recently, Zhang et al., reported a nuclear function of STING that activates the transcription factor AHR, primarily involved in homeostasis between the gut and microbiota and metabolism [35]. Whether the same pathway contributes to the STING-induced early response of ECs to IR remains to be explored. However, our findings of the presence of STING in the nuclei 24 h after IR in conjunction with the down-regulation of metabolism-related genes (*CYP26B1* and *CYP2S1*) 72 h after IR supports this. Conversely, 72 h after IR, we observed an accumulation of STING in peri-nuclear punctate structures and a significant up-regulation of cGAS-STING pathway target genes, including *IFIT1*, *MX1*, *MX2*, and *OAS2*.

While 24 h after IR of HUVECs, we observed significant changes in the activation of NF-κβ regardless of the IR dose, the proportion of NF-κβ positive nuclei at 72 h after IR was significantly higher after 2 Gy oF IR, and not after 5 Gy of IR comparing with the control, suggesting that IR exerts a more pronounced impact on NF-κβ at earlier timepoints. Consistent with these results, we observed IR-induced nuclear translocation of NF-κβ in TECs in vivo after 5 × 5 Gy of IR, at 24 and 48 h after IR.

Altogether, and considering NF-κβ as one of the main targets of cGAS-STING signaling, we propose that IR-induced DNA damage in ECs and TECs activates STING in a time-dependent manner; nuclear STING signaling predominates at 24 h after IR, whereas cytoplasmic cGAS-STING signaling becomes dominant after 48 h. The importance of cGAS-STING pathway in ECs has recently been demonstrated in lung ischemia–reperfusion injury after lung transplantation where its activation was induced by mitochondrial DNA and resulted in pyroptosis of endothelial cells [47], which could be ameliorated using inhibitors of cGAS and STING. Moreover, Verhoeven et al. [48] showed that the loss of autophagy in TECs in melanoma increases cytoplasmic DNA levels, which can lead to the transcriptional expression of an immunostimulatory/inflammatory TEC phenotype driven by increased NF-kB and STING signaling. However, further research with more precise biochemical and molecular approaches is required to fully elucidate these mechanisms in TECs after IR.

Previous studies have shown that both senescent cells, in general, and irradiated endothelial cells secrete a variety of cytokines that can influence their environment [49,50,51,52]. Similarly, our data suggests a time- and dose-dependent up-regulation of ECM-related signaling pathways in response to NF-κβ-induced chemokine and cytokine expression after IR in HUVECs. We observed IR-induced up-regulation of cell adhesion, ECM–receptor interactions, and focal adhesion signaling pathways. Selectins (SELE, SELP) and cell adhesion molecules (ICAM-1, VCAM-1) play a crucial role in immune cell rolling, interactions with ECs, and subsequent extravasation of immune cells in vivo [9,20]. Among them, although only *SELE* was significantly up-regulated when comparing the irradiated with the control samples, the transcript levels of *SELE*, *SELP*, and *ICAM-1* increased significantly 72 h after IR compared with 24 h after IR, suggesting that the main ECM changes induced by IR occur at later timepoints. At the protein level, we observed a significant increase in VCAM-1 expression on the cell membrane of irradiated HUVECs, regardless of IR dose and time after exposure, confirming that IR triggers a cascade of events leading to EC activation.

The observed IR-induced activation of TECs provide important entry points for immune cell infiltration, which are often not sufficient to achieve tumor eradication. However, a potentially synergistic effect could be achieved when combined with STING agonists, especially when these are administered systemically and target the TECs that are readily accessible via the bloodstream. This combination could further increase the frequency of activated TECs in tumors, creating additional pathways for immune cells to enter tumors. STING agonists have already been shown to be effective in increasing the infiltration of T cells and other immune effectors into the tumor, but they have not yet been used to increase the frequency of TECs. Particularly in immunologically deserted tumors, this approach could also prove synergistic with immune checkpoint inhibitors such as anti-PD-1 or anti-CTLA-4 antibodies, which have been shown to be effective in certain cancers in combination with RT. However, the timing of the different treatment modalities would need to be precisely determined, as the killing of immune cells by IR would interfere with the beneficial effects of increasing the frequency of activated TECs [53,54,55].

Our study also has some limitations. The main part of the study was only conducted in vitro. Therefore, it was not possible to assess whether the observed IR-induced endothelial activation and changes in ECM-related signaling pathways lead to enhanced infiltration of immune cells into the tumor and subsequent activation of an anti-tumor immune response. This limitation could be addressed in the future by using perfusable vasculature-on-a-chip models [56] that could be perfused with specific populations of immune cells, which would allow them to be tracked in real time and follow their interaction with activated ECs after IR. Alternatively, the profiling of tumor infiltrating immune cells using multiplex spatial tissue analysis would provide accurate information about which immune cells and when they interact with the IR-activated TECs and whether the IR-activated TECs are their site of entry into the tumor. Furthermore, our in vitro study was performed on normal, commercially available EC lines and not on TECs. Although it is now possible to isolate TECs from tumors [5,27,57], it is still not possible to perform large-scale experiments with them. The addition of longer timepoints after IR and other doses of IR could contribute to a deeper understanding of the temporal sequence of the observed effects and whether these are maintained at higher IR doses. Lastly, the observed modulation of the cGAS-STING pathway should be further confirmed using knock-out cell lines and inhibitors of the key proteins to elucidate the exact mechanisms governing the response of ECs to IR. However, this was beyond the scope of this manuscript.

## 5. Conclusions

In conclusion, it is now generally accepted that RT has immunomodulatory effects on the tumor and surrounding tissue involving multiple components, such as immune cells, the extracellular matrix, endothelial and epithelial cells. However, the full extent of these changes and, in particular, the specific contributions of the different components are still unclear [53,58,59,60]. Our study provides important insights into how different doses of IR doses induce complex changes in ECs and TECs, including changes in proliferation, cell cycle regulation, and expression of inflammatory and adhesion molecules. Our study is the first detailed RNA seq-based transcriptomic study on HUVECs after clinically relevant IR doses (2 Gy, 5 Gy) at two timepoints (24 h, 72 h) and links the observed up-regulation of immune and ECM remodeling pathways with an increase in STING and NF-κB activation in vitro and in vivo. Our results suggest that IR not only suppresses EC proliferation but also activates ECs and TECs in a dose- and time-dependent manner, possibly enhancing immune cell recruitment and potentially promoting anti-tumor immune responses. Our research thus provides fundamental insights into how IR affects the function and activation of ECs and TECs, which could contribute to the activation of the anti-tumor immune response. Based on this knowledge, RT regimens could be optimized to exploit the potential benefit of TEC-related activation of the anti-tumor immune response and improve the efficacy of RT.

## Data Availability

The data supporting the findings of this research are available from the corresponding author upon reasonable request. Whole transcriptome RNA sequencing data used in this research are publicly available in the NCBI GEO repository under the accession number GSE287078.

## Supplementary Materials

The following supporting information can be downloaded at https://www.mdpi.com/article/10.3390/cancers17172842/s1, Figure S1: Proliferation and morphology of murine and human EC lines after 6 Gy IR.; Figure S2: Comparison of global transcriptomic profiles of irradiated HUVECs.; Figure S3: Top 20 differentially enriched pathways in irradiated HUVECs compared with the control.; Figure S4: Expression levels of *SELE* (a), *TNFSF4* (b), and *CXCL12* (c) after IR with 2 Gy and 5 Gy, measured by qRT-PCR as a validation of RNA sequencing results.; Figure S5: IR leads to down-regulation of cell cycle and up-regulation of immune response-related pathways in TECs; Table S1: The impact of 2 Gy of IR on the regulation of signaling pathways in HUVECs.; Table S2: The effect of IR with 5 Gy on the regulation of signaling pathways in HUVECs.; Table S3: The impact of 2 or 5 Gy of IR on the cell cycle-related genes in HUVECs.; Table S4: The impact of time after the IR with 2 or 5 Gy on cell cycle-related genes in HUVECs. Table S5: Changes in the expression of ECM-associated genes after 2 or 5 Gy of IR in HUVECs.; Table S6: Time-dependent changes in ECM-associated genes after IR with 2 or 5 Gy in HUVECs. Table S7: The effect of 2 or 5 Gy IR on the immune response-associated genes in HUVECs; Table S8: Time-dependent changes in immune response-related genes after IR with 2 or 5 Gy in HUVECs. Table S9. Top 15 significantly up- and down-regulated pathways in TECs in vivo at 48 h after 15 Gy of IR.

## Abbreviations

The following abbreviations are used in this manuscript:

*ADAM10*	ADAM Metallopeptidase Domain 10
*AHR*	Aryl Hydrocarbon Receptor
AM	Arithmetic mean
ARRIVE	Animal Research: Reporting of In Vivo Experiments
ATCC	American Type Culture Collection
*AURKB*	Aurora Kinase B
*BUB1*	Mitotic Checkpoint Serine/Threonine Kinase
*BUB1B*	Mitotic Checkpoint Serine/Threonine Kinase B
*CCNA2*	Cyclin A2
*CDC6*	Cell Division Cycle 6
*CDKN1A*	Cyclin Dependent Kinase Inhibitor 1A
*CDKN2C*	Cyclin Dependent Kinase Inhibitor 2C
*CDT1*	Chromatin Licensing And DNA Replication Factor 1
CR	Complete response
*CXCL1*	C-X-C Motif Chemokine Ligand 1
*CXCL11*	C-X-C Motif Chemokine Ligand 11
*CXCL12*	C-X-C Motif Chemokine Ligand 12
*CXCL2*	C-X-C Motif Chemokine Ligand 2
*CXCL8*	C-X-C Motif Chemokine Ligand 8
*CXCR4*	C-X-C Motif Chemokine Receptor 4
*CYP26B1*	Cytochrome P450 Family 26 Subfamily B Member 1
*CYP2S1*	Cytochrome P450 Family 2 Subfamily S Member 1
DE	Differentially expressed
DGE	Differential gene expression
*E2F1*	E2F Transcription Factor 1
EC	Endothelial cells
ECM	Extracellular matrix
*EMP2*	Epithelial Membrane Protein 2
FBS	Fetal bovine serum
FC	Fold change
FDR	False discovery rate
GSEA	Gene Set Enrichment analysis
HBSS	Hanks’ Balanced Salt Solution
HUVECs	Human umbilical vein endothelial cells
ICAM-1	Intercellular adhesion molecule 1
*IFIT1*	Interferon Induced Protein With Tetratricopeptide Repeats 1
*IL-12A*	Interleukin 12a
*IL-1B*	Interleukin 1 Beta
*IL-6*	Interleukin 6
*IL-68*	Interleukin 68
IR	Irradiation
*Irf7*	Interferon Regulatory Factor 7
*ITGA10*	Integrin Subunit Alpha 10
*JAG1*	Jagged Canonical Notch Ligand 1
KEGG	Kyoto Encyclopedia of Genes and Genomes
*LAMC2*	Laminin Subunit Gamma 2
*MDM2*	MDM2 Proto-Oncogene
mRNA	Messenger RNA
*MX1*	MX Dynamin Like GTPase 1
*MX2*	MX Dynamin Like GTPase 2
*MYLK2*	Myosin Light Chain Kinase 2
NES	Nuclear factor kappa B
NPDXE	Novartis Institutes for BioMedical Research PDX encyclopedia
*OAS2*	2′-5′-Oligoadenylate Synthetase 2
OCT	Optimal Cutting Temperature (compound)
*PARD6G*	Par-6 Family Cell Polarity Regulator Gamma
PBS	Phosphate-buffered saline
PCA	Principal component analysis
PD	Progressive disease
PECAM-1	Platelet and endothelial cell adhesion molecule 1
PFA	Paraformaldehyde
PI	Propidium iodide
*PLAT*	Plasminogen activator
*PLCB2*	Phospholipase C Beta 2
*PML*	PML Nuclear Body Scaffold
PR	Partial response
qRT-PCR	Quantitative real-time polymerase chain reaction
*RigI*	RNA Sensor RIG-I
ROS	Reactive oxygen species
RT	Radiotherapy
SD	Stable disease
SDC1	Syndecan 1
SELE	Selectin E
SELP	Selectin P
SEM	Standard error of the mean
*SORBS1*	Sorbin And SH3 Domain Containing 1
*SPRY4*	Sprouty homolog 4
*STING*	Stimulator Of Interferon Response CGAMP Interactor 1
TEC	Tumor endothelial cell
TME	Tumor microenvironmet
*TNFSF4*	TNF Superfamily Member 4
*TNFSF18*	TNF Superfamily Member 18
TNFα	Tumor necrosis factor alpha
*TRAF1*	TNF Receptor Associated Factor 1s
*TUBA1B*	Tubulin Alpha 1b
*TUBB4B*	Tubulin Beta 4B Class IVb
VCAM-1	Vascular cell adhesion molecule 1
VE-Cad	Vascular Endothelial Cadherin
WGA	Wheat germ agglutinin
