# Supplementary material for: Time- and Dose-Dependent Effects of Irradiation on Endothelial and Tumor Endothelial Cells: Transcriptional, Molecular, and Functional Changes Driving Activation In Vitro and In Vivo
Source: Cancers (Basel). 2025 Aug 29;17(17):2842. doi: 10.3390/cancers17172842 (PMC12427429; doi:10.3390/cancers17172842)
Supplement: Supplementary file 1 [file cancers-17-02842-s001.zip › cancers-3778516-supplementary.pdf]

Article

Time- and Dose-Dependent Effects of Irradiation on Endothelial and Tumor Endothelial Cells: Transcriptional, Molecular, and Functional Changes Driving Activation In Vitro and In Vivo

Iva Santek [1,2], Gregor Sersa [1,3] and Bostjan Markelc [1,4,*]

1 Department of Experimental Oncology, Institute of Oncology Ljubljana, Zaloska cesta 2, 1000 Ljubljana, Slovenia; isantek@onko-i.si (I.S.); gsersa@onko-i.si (G.S.)
2 Faculty of Medicine, University of Ljubljana, Vrazov trg 2, 1000 Ljubljana, Slovenia
3 Faculty of Health Sciences, University of Ljubljana, Zdravstvena pot 5, 1000 Ljubljana, Slovenia
4 Biotechnical Faculty, University of Ljubljana, Jamnikarjeva ulica 101, 1000 Ljubljana, Slovenia
* Correspondence: bmarkelc@onko-i.si (B.M.)

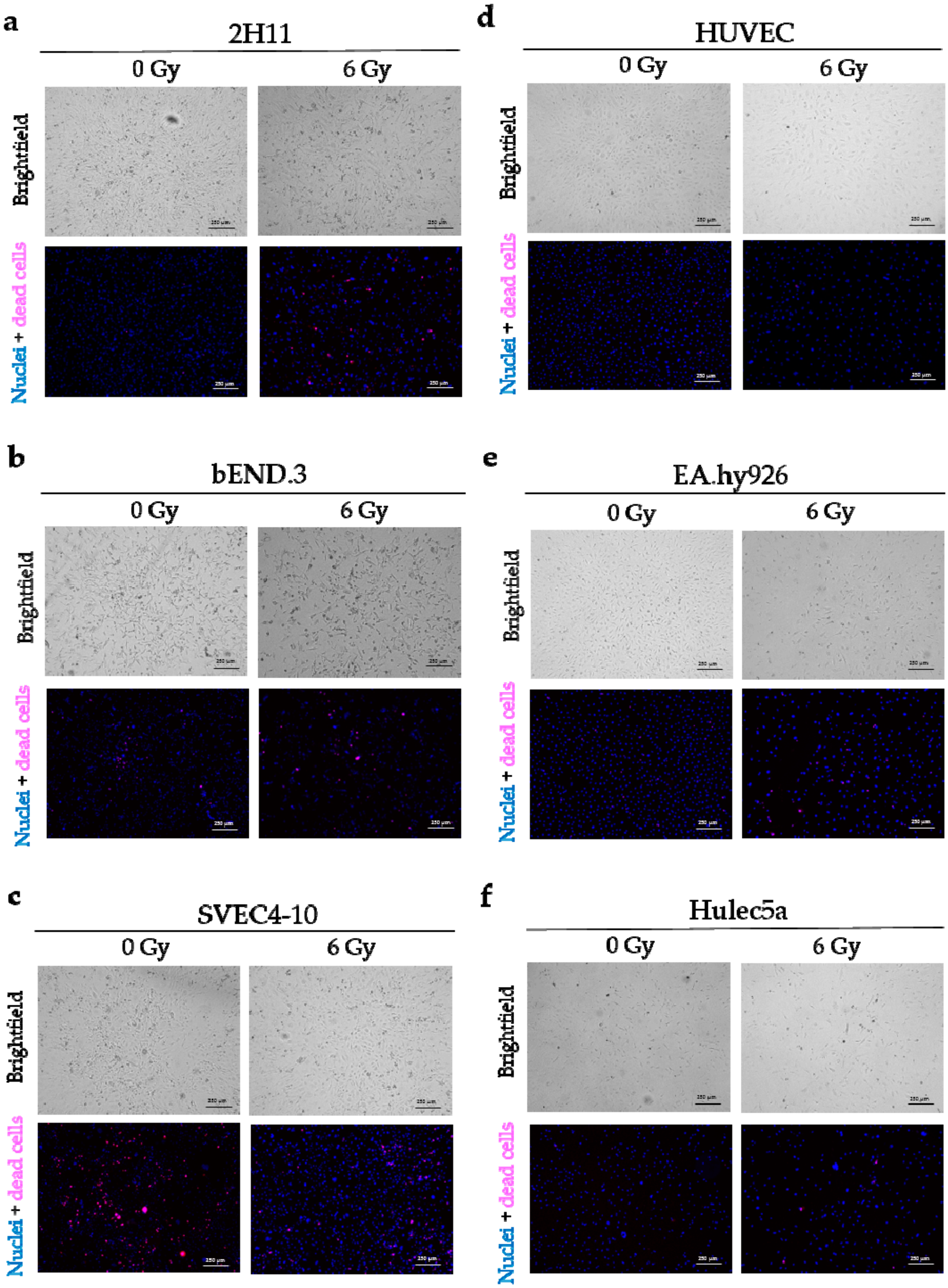

Supplementary Figure S1. Proliferation and morphology of murine and human EC lines after 6 Gy IR. Representative images of control (0 Gy) ECs and ECs irradiated with 6 Gy at 72 h were captured using Cytation 1 Cell Imaging Multi-Mode Reader. Hoechst 33342 staining (blue) was used to visualize all nuclei, while PI (purple) selectively stained the nuclei of dead cells. **a-c** Murine EC lines 2H11 (**a**), bEND.3 (**b**), and SVEC4-10 (**c**). **d-f** Human EC lines HUVEC (**d**), EA.hy926 (**e**), and Hulec5a (**f**). *n* = 3. Brightfield and fluorescence images were captured from

the same microscopic field. Quantification is presented on Figures 1 and 2. scale bar = 250μm. PI – Propidium Iodide. IR – irradiation.

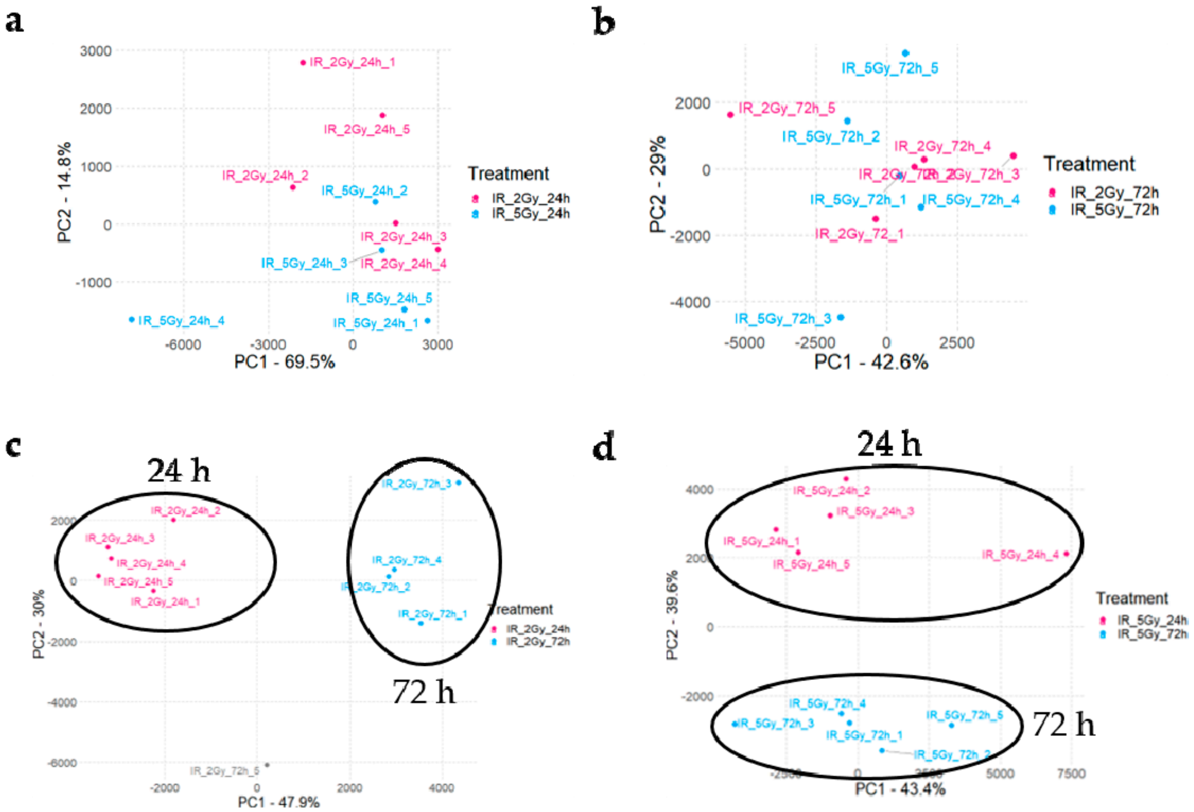

Supplementary Figure S2. Comparison of global transcriptomic profiles of irradiated HUVECs**.** HUVEC cells were irradiated with 2 or 5 Gy followed by RNA isolation and sequencing at 24 or 72 h after IR. Global transcriptomic profiles of irradiated HUVECs were analyzed in comparison to their respective controls and among experimental groups (varying doses and time points). PCA plots depict normalized gene expression levels. **a-b** PCA plots presenting dose-dependent effects on transcriptomes of irradiated HUVECs, revealing no distinct clustering between samples analyzed 24 h (**a**) and 72 h (**b**) after IR. c-d PCA plots showing the time-dependent effects on the transcriptomes of irradiated HUVECs, presenting clustering of samples based on time points after IR with 2 Gy (**c**) or 5 Gy (**d**). *n* = 5. PCA–principal component analysis. PC1 - principal component 1, PC2 - principal component 2. IR–irradiation.

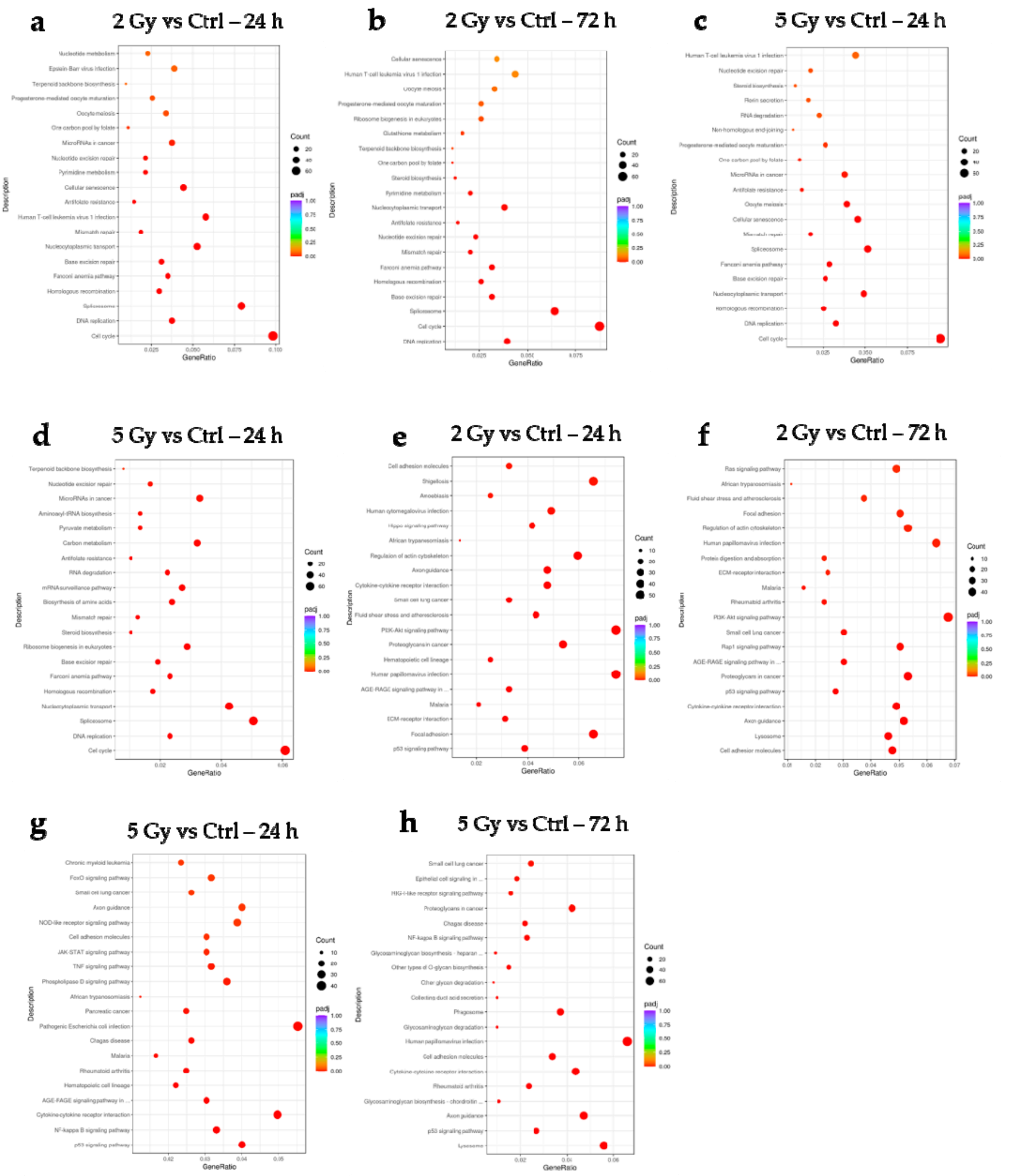

Supplementary Figure S3. Top 20 differentially enriched pathways in irradiated HUVECs compared to control. **a-d** Top 20 significantly down-regulated pathways identified in samples analyzed at 24 or 72 h after IR with 2 Gy (**a, b**) or 5 Gy (**c, d**). **e-h** Top 20 significantly up-regulated pathways identified in samples analyzed 24 or 72 h after IR with 2 Gy (**e, f**) or 5 Gy (**g, h**). Pathway enrichment was evaluated using KEGG analysis. Counts represent the number of differentially expressed genes from the gene list associated with a particular KEGG pathway. GeneRatio represents the proportion of differentially expressed genes in a particular pathway and all differentially expressed genes found in KEGG database. Pathways were considered to be significantly enriched if the adjusted P-value < 0.05. n = 5. Padj – Adjusted P-Values. KEGG - Functional Kyoto Encyclopedia of Genes and Genomes.

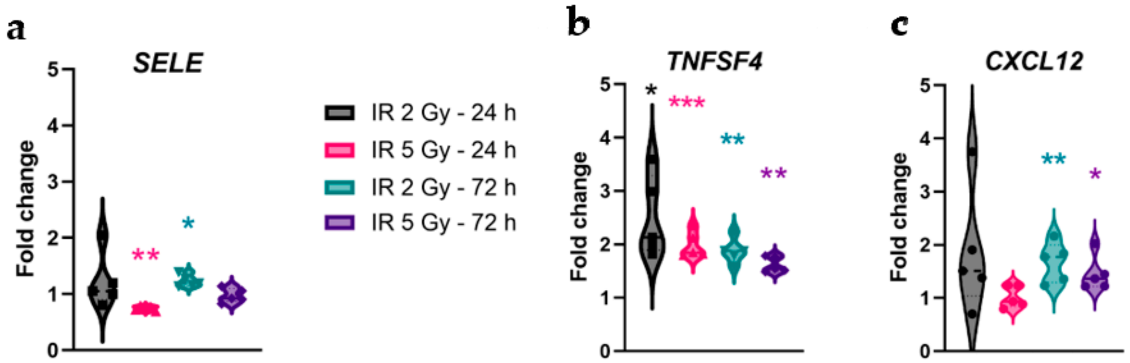

Supplementary Figure S4. Expression levels of *SELE* (**a**), *TNFSF4* (**b**), and *CXCL12* (**c**) at 24 h or 72 h after IR with 2 Gy and 5 Gy, measured by qRT-PCR as validation of RNA sequencing results. Statistical significance was determined by unpaired t-test for normally distributed data. $n = 5$ per group. Statistical significance shown as $p < 0.05$ (*), $p < 0.01$ (**), $p < 0.001$ (***), and $p < 0.0001$ (****). IR – Irradiation. *SELE* - Endothelial adhesion molecule 1. *TNFSF4* - Tumor necrosis factor ligand superfamily member 4. *CXCL12* - CXC motif chemokine 12.

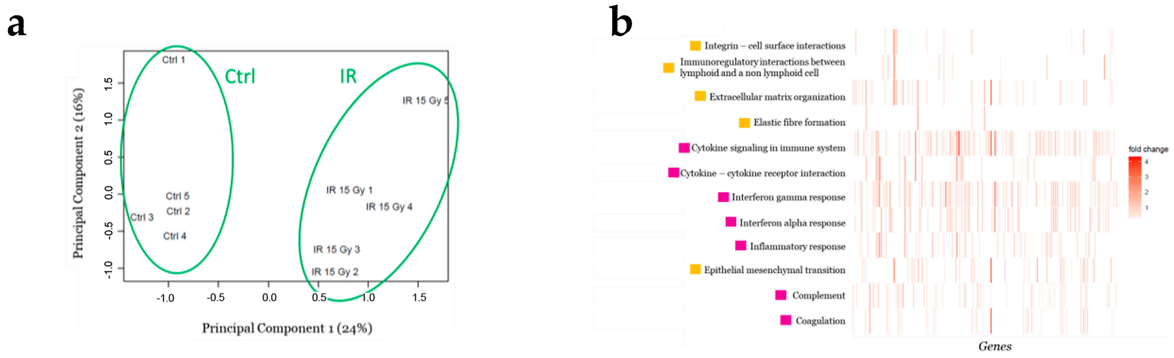

Supplementary Figure S5. IR leads to down-regulation of cell cycle and up-regulation of immune response-related pathways in TECs. To elucidate the transcriptomic background underlying the IR-induced changes in the expression of TEC activation proteins observed in vivo, we analyzed a publicly available RNA sequencing dataset of TECs isolated from MC38 murine colon carcinomas (GSE168481). A single dose of 15 Gy of IR was delivered to the tumors while mice anesthetized with isoflurane were restrained in a lead shield, leaving only the tumors exposed to the IR [7,27]. Irradiated samples (48 h after IR with 15 Gy) were compared to non-irradiated controls. PCA analysis showed the global transcriptomic profiles of irradiated TECs clustered distinctly from non-irradiated controls(**a**). DGE analysis identified 769 significantly differentially expressed genes (236 down-regulated and 533 up-regulated) in the irradiated samples compared to controls. GSEA demonstrated 44 significantly up-regulated, and 101 significantly down-regulated pathways at 48 h after 15 Gy of IR. Interestingly, among the top 15 up-regulated pathways, 10 were associated with immune responses, including IFN*α* and IFN*γ* responses, cytokine–cytokine receptor interactions, Inflammatory response, and TNF*α* signaling via NF-κB. Contrary, among the top 15 down-regulated pathways, 13 were related to cell cycle inhibition, including E2F targets, G2M checkpoint, and M phase (Supplementary Table 9). We next focused on TEC activation-related pathways that were significantly up-regulated in the irradiated samples. We categorized these pathways into two groups: ECM alteration and immune response-associated pathways (**b**). RNA sequencing analysis revealed several up-regulated genes commonly expressed across these pathways, including *Icam1*, *Vcam1*, *Cxcl10*, *Il6*, and *Il15*, suggesting their involvement in multiple processes during TEC activation. The IR-induced up-regulation of ECM-related pathways, such as Integrin–cell surface interactions, ECM organization, and Elastic fiber formation, were characterized by increased expression of *Mmp2*, *Col1a1*, *Thbs1*, and *Itgb3*, while immune-associated pathways were marked by the over-expression of genes including *Mx1*, *Usp18*, *Cxcl11*, *Ccl2*, and *Il15*. (**b**). To further investigate IR-induced DNA sensing in TECs, which we also observed in irradiated samples in vitro, we examined the expression of key DNA sensors. At 48 h after IR, TECs exhibited significant up-regulation of genes involved in DNA sensing, including *Zbp1*, *Ddx58*, and *Tmem173*, which are directly implicated in STING signaling. Altogether, these findings are consistent with our in vitro results on irradiated HUVECs, further supporting the role of TECs and ECs in modulating the IR-induced immune response and suggesting the importance of the STING signaling in the response of ECs and TECs to IR. n = 5.IR. irradiation. TECs–tumor endothelial cells. PCA–principal component analysis. DGE–differential gene expression. GSEA–Gene Set Enrichment Analysis. See also Figure 7.

	2 Gy - 24 h vs control VS 2 Gy - 72 h vs control						
	Only in 24 h	Adjusted P-Value	Only in 72 h	Adjusted P-Value	Common	Adjusted P-Value (24 h after IR)	Adjusted P-Value 72 h after IR)
Up-regulated	Hematopoietic cell lineage	0.002	Lysosome	0.046	p53 signaling pathway	240E-10	9.70E-04
	ECM-receptor interaction	0.009			Cell adhesion molecules	0.,033	0.003
	PI3K-Akt signaling pathway	0.012			Small cell lung cancer	2.09E-05	0.012
	Cytokine-cytokine receptor interaction	0.003					
	NF-kappa B signaling pathway	0.003					
	Malaria	5.80E-05					
	AGE-RAGE signaling pathway in diabetic complications	0.004					
	Human papillomavirus infection	0.001					
	African trypanosomiasis	0.033					
	Pancreatic cancer	0.033					
	Rheumatoid arthritis	0.034					
	Basal cell carcinoma	0.033					
	Inflammatory bowel disease	0.033					
	Toxoplasmosis	0.034					
Down-regulated	MicroRNAs in cancer	4.90E-04			Cell cycle	8.75E-19	1.35E-10
	Antifolate resistance	0.001			DNA replication	1.23E-10	1.15E-11
	Nucleocytoplasmic transport	0.007			Homologous recombination	1.36E-05	0.001
	Human T-cell leukemia virus 1 infection	0.003			Spliceosome	1.23E-04	0.046
	Epstein-Barr virus infection	0.010			Fanconi anemia pathway	9.60E-05	0.005
	Transcriptional misregulation in cancer	0.020			Cellular senescence	1.30E-04	0.046
	Nucleotide excision repair	0.023			Base excision repair	4.50E-04	0.001
	Viral carcinogenesis	0.033			Pyrimidine metabolism	0.090	0.005
	One carbon pool by folate	0.038			Mismatch repair	0.010	0.005
	Nucleotide metabolism	0.033					

Supplementary Table S1. The impact of 2 Gy of IR on the regulation of signaling pathways in HUVECs. Significantly enriched pathways identified by KEGG analysis shared between samples analyzed at 24 and 72 h after IR with 2 Gy (in comparison to their corresponding controls). Enrichment was considered statistically significant for Adjusted P-values < 0.05. GeneRatio > 1 and GeneRatio < 1 were chosen as thresholds for up-regulation and down-regulation of a pathway, respectively. *n* = 5. IR-irradiation. KEGG - Functional Kyoto Encyclopedia of Genes and Genomes.

	5 Gy - 24 h vs control VS 5 Gy - 72 h vs control						
	Only in 24 h	Adjusted P-value	Only in 72 h	Adjusted P-value	Common	Adjusted P-Value (24 h after IR)	Adjusted P-Value (72 h after IR)
Up-regulated	NF-kappa B signaling pathway	0.003	Cytokine-cytokine receptor interaction	0.026	Small cell lung cancer	0.003	0.007
	Viral protein interaction with cytokine and cytokine receptor	0.017	Lysosome	1.07E-09	Rheumatoid arthritis	0.011	0.003
	Hematopoietic cell lineage	0.007	Glycosaminoglycan biosynthesis - chondroitin sulfate / dermatan sulfate	0.017	p53 signaling pathway	7.59E-11	2.86E-06
	Th17 cell differentiation	0.023	Axon guidance	0.017			
	TNF signaling pathway	0.045					
	Malaria	0.003					
	AGE-RAGE signaling pathway in diabetic complications	0.005					
	Transcriptional misregulation in cancer	0.007					
	Gastric cancer	0.013					
	Colorectal cancer	0.020					
	Pancreatic cancer	0.025					
Down-regulated	Cellular senescence	5.59E-05	Glycosaminoglycan degradation	0.019	Fanconi anemia pathway	0.003	0.0017
	Base excision repair	0.002	PPAR signaling pathway	0.017	MicroRNAs in cancer	0.005	5.29E-05
	Mismatch repair	0.015			DNA replication	3.66E-09	8.08E-06
	Antifolate resistance	0.002			Cell cycle	1.37E-16	4.62E-07
	Human T-cell leukemia virus 1 infection	0.003			Homologous recombination	7.70E-05	0.025
	Nucleocytoplasmic transport	0.011					
	Renin secretion	0.011					

Supplementary Table S2. The effect of IR with 5 Gy on the regulation of signaling pathways in HUVECs. Significantly enriched pathways identified by KEGG analysis shared between samples analyzed at 24 and 72 h after 5 Gy of IR (compared to their corresponding controls). Enrichment was considered statistically significant for Adjusted P-values < 0.05. GeneRatio > 1 and GeneRatio < 1 were determined as thresholds for up-regulation and down-regulation of a pathway, respectively. $n = 5$. IR - irradiation. KEGG - Functional Kyoto Encyclopedia of Genes and Genomes.

24 h after IR						72 h after IR					
2 Gy vs control			5 Gy vs control			2 Gy vs control			5 Gy vs control		
Gene	log_2FC	Adjusted P-value	Gene	log_2FC	Adjusted P-value	Gene	log_2FC	Adjusted P-value	Gene	log_2FC	Adjusted P-value
AURKB	-2.9046	5.30E-105	AURKB	-4.002073115	5.33E-121	AURKB	-1.379107	1.44E-10	AURKB	-3.45209925	4.45E-100
BUB1	-2.7126	1.27E-125	BUB1	-3.320947717	2.85E-129	BUB1	-1.302442	8.81E-26	BUB1	-3.359151592	1.34E-219
BUB1B	-2.8459	2.20E-127	BUB1B	-3.761764344	7.94E-124	BUB1B	-1.450682	5.95E-40	BUB1B	-3.235286694	4.26E-186
CCNA2	-2.5548	1.25E-184	CCNA2	-3.356566877	3.68E-160	CCNA2	-1.237858	7.51E-33	CCNA2	-2.965576599	1.21E-240
CCNB1	-2.0635	1.76E-147	CCNB1	-2.3663565	3.48E-129	CCNB1	-1.122427	7.20E-33	CCNB1	-2.369617709	6.99E-209
CCNB2	-2.6035	2.87E-96	CCNB2	-3.221722565	3.55E-113	CCNB2	-1.48065	6.58E-28	CCNB2	-3.01695857	5.95E-143
CCNE2	-1.5439	1.99E-08	CCNE2	-2.135484089	2.27E-12	CCND2	1.3171565	8.63E-15	CCND2	1.832943921	5.96E-42
CDC20	-2.752	7.13E-174	CDC20	-3.30507656	7.17E-199	CDC20	-1.367784	6.19E-30	CCNE2	-1.45705656	1.00E-08
CDC25A	-1.0825	1.44E-16	CDC25A	-1.168183549	9.65E-22	CDC25A	-0.610133	0.000322257	CDC20	-3.44512033	8.80E-221
CDC25B	-0.8341	5.32E-41	CDC25B	-0.671165398	4.90E-26	CDC25B	-0.716072	8.84E-15	CDC25A	-1.299395893	3.21E-34
CDC25C	-3.2037	2.02E-31	CDC25C	-3.596399932	1.54E-32	CDC25C	-1.616133	2.94E-20	CDC25B	-1.000685716	2.02E-61
CDC45	-2.3986	9.53E-57	CDC45	-2.612561173	1.96E-72	CDC45	-1.283486	1.34E-19	CDC25C	-3.55489978	6.25E-49
CDC6	-1.9879	2.89E-65	CDC6	-2.522318022	1.56E-60	CDC6	-1.118622	1.60E-32	CDC45	-3.256272744	1.09E-104
CDC7	-0.7866	0.000000107	CDC7	-1.010134866	5.10E-12	CDCA5	-1.222861	2.13E-23	CDC6	-2.785274078	3.27E-149
CDCA5	-2.2928	1.39E-96	CDCA5	-2.776759846	1.08E-104	CDK1	-1.159042	4.57E-30	CDC7	-1.062992463	2.70E-11
CDK1	-2.2756	3.77E-70	CDK1	-2.962338426	5.90E-115	CDKN1A	1.0378339	1.00E-22	CDCA5	-2.45794855	1.11E-106
CDKN1A	1.3454	3.73E-120	CDKN1A	1.808519897	1.49E-139	CDKN2C	-1.603744	4.61E-16	CDK1	-2.787498435	8.30E-194
CDKN2C	-2.1217	5.98E-49	CDKN2C	-2.345125502	2.55E-44	CDT1	-1.086361	2.79E-10	CDKN1A	1.428761745	1.51E-223
CDKN2D	-0.6815	0.003749297	CDT1	-2.007479611	4.66E-40	CXCL8	0.8711012	1.34E-08	CDKN2C	-3.114786741	7.30E-77
CDT1	-1.9185	1.49E-38	DBF4	-1.192785028	5.30E-24	DBF4	-0.784062	1.35E-05	CDT1	-2.47868012	3.20E-83
DBF4	-0.8286	2.50E-13	DBF4B	-0.823593298	1.47E-07	DBF4B	-0.627546	2.32E-06	CXCL8	1.102786241	3.06E-35
DBF4B	-1.2278	8.05E-16	DDX11	-0.585550783	1.92E-08	DDX12P	-1.282136	0.000191467	DBF4	-1.349763848	7.44E-41
DDX12P	-1.8863	0.000000149	DDX12P	-1.16542161	0.003808278	DNA2	-0.946288	1.22E-08	DBF4B	-1.523091439	7.66E-33
DNA2	-1.1425	8.10E-14	DNA2	-1.445972947	2.23E-19	E2F1	-0.931179	3.56E-08	DDX11	-0.818569155	1.74E-11
E2F1	-2.1563	1.74E-43	E2F1	-2.607003377	2.42E-52	E2F2	-1.256732	0.000195587	DDX12P	-1.481986	3.00E-06
E2F2	-2.645	7.46E-09	E2F2	-4.51836163	2.40E-11	ESCO2	-1.086475	3.79E-08	DNA2	-1.333876286	1.55E-17
ESCO2	-2.6547	8.16E-49	ESCO2	-3.282828403	6.46E-58	ESPL1	-1.233794	2.33E-24	E2F1	-2.351439423	2.96E-56
ESPL1	-2.9872	4.66E-92	ESPL1	-2.991377685	1.30E-88	FBXO5	-0.887823	2.49E-11	E2F2	-2.995576156	8.52E-17
FBXO5	-1.5121	3.59E-27	FBXO5	-1.843443922	2.03E-37	FEN1	-0.832257	1.04E-14	ESCO2	-3.068904165	4.67E-78
FEN1	-1.2631	8.16E-64	FEN1	-1.443051494	3.88E-52	FOXM1	-1.407088	1.24E-50	ESPL1	-2.769581319	7.88E-94
FOXM1	-2.4394	9.93E-198	FOXM1	-2.43578375	5.96E-178	HMGB1P6	-0.609412	1.12E-05	FBXO5	-1.900644332	2.46E-47
IL6	0.6018	0.003131212	GADD45A	0.6409032	1.47E-16	KNL1	-1.38639	6.62E-14	FEN1	-1.415808258	3.44E-77
KNL1	-2.7107	7.00E-114	IL6	0.643824661	0.001689589	LIN9	-0.658637	0.002298268	FOXM1	-3.289167698	3.15E-237
LIN9	-0.6274	0.000444106	KNL1	-3.342390572	2.32E-110	MAD2L1	-0.876057	6.05E-21	GADD45A	0.789441308	4.86E-23
MAD2L1	-1.6483	1.60E-40	LIN9	-1.084864111	1.63E-08	MCM2	-0.797719	8.71E-07	HMGB1P5	-0.63829753	4.37E-13
MCM2	-1.425	1.14E-52	MAD2L1	-2.201460858	1.23E-63	MCM4	-0.703718	2.22E-23	HMGB1P6	-0.778321009	2.27E-22
MCM3	-0.638	4.56E-29	MCM2	-1.453745677	9.45E-40	MCM5	-0.911869	5.04E-16	KNL1	-3.027531719	6.08E-136
MCM4	-1.3643	1.10E-72	MCM3	-0.675123543	1.37E-28	MCM6	-0.665568	1.13E-18	LIN9	-1.162447718	8.02E-12
MCM5	-1.1134	5.18E-40	MCM4	-1.682478484	3.42E-102	MCM7	-0.685886	2.12E-12	MAD2L1	-2.239527787	5.82E-99
MCM6	-0.7253	2.49E-21	MCM5	-1.013270302	5.03E-25	MDM2	0.7742395	5.13E-06	MCM2	-1.646547462	6.50E-57
MCM7	-0.9372	1.74E-55	MCM6	-0.965143254	5.29E-41	MTBP	-0.877134	2.55E-06	MCM3	-0.851747564	2.42E-40
MDM2	1.2203	1.75E-68	MCM7	-0.921855274	1.60E-43	MYBL2	-1.395375	1.11E-23	MCM4	-1.794822981	1.76E-197
MTBP	-0.7842	0.000531413	MDM2	1.557512561	5.28E-84	NDC80	-1.488824	2.53E-36	MCM5	-1.570402651	1.24E-66
MYBL2	-2.73	4.89E-199	MTBP	-0.948943131	0.000183392	NEIL3	-0.896156	4.29E-05	MCM6	-1.203055478	2.90E-65
NDC80	-2.7616	1.12E-82	MYBL2	-3.240507946	2.95E-228	ORC1	-0.851444	2.31E-07	MCM7	-1.257211427	2.15E-47
NEIL3	-2.1458	1.22E-16	NDC80	-3.793835669	1.15E-106	ORC6	-0.668244	1.04E-05	MDM2	1.017492174	2.79E-44
ORC1	-1.9244	1.52E-19	NEIL3	-3.4930177	6.16E-26	PKMYT1	-1.071081	1.06E-08	MTBP	-1.274673031	2.91E-13
ORC6	-1.6209	1.39E-30	ORC1	-2.694748186	1.11E-30	PLK1	-1.385228	2.45E-34	MYBL2	-3.375208162	6.07E-292
PKMYT1	-2.333	7.57E-37	ORC6	-2.077933353	3.86E-45	POLE2	-0.825169	5.33E-05	NDC80	-3.287484389	1.47E-163
PLK1	-2.8722	8.84E-138	PARP2	-0.586975837	7.50E-06	PRIM1	-0.992961	4.93E-05	NEIL3	-2.979834454	1.85E-37
POLA2	-0.7068	0.008749593	PKMYT1	-3.389827966	4.69E-43	PTTG1	-1.25422	6.07E-33	ORC1	-2.789948077	6.09E-56
POLD1	-0.6629	0.000000136	PLK1	-3.52555045	2.34E-148	RFC3	-0.60225	0.014052992	ORC6	-1.87762337	7.96E-44
POLE	-0.7362	1.71E-11	POLA1	-0.737769273	2.54E-06	RFC4	-0.746867	4.00E-09	PARP1	-0.672458166	5.28E-52
POLE2	-1.1555	0.000000182	POLE	-0.828293146	1.79E-14	SGO1	-1.235491	2.07E-05	PARP2	-0.651883689	1.73E-13
PRIM1	-1.5948	6.38E-12	POLE2	-1.370734689	4.20E-08	TICRR	-1.167361	6.59E-13	PKMYT1	-3.086757928	8.20E-70
PTTG1	-1.6909	4.93E-50	PRIM1	-1.755642454	7.38E-10	TRIP13	-1.057815	1.04E-20	PLK1	-3.309856011	7.24E-293
RBL1	-0.9199	1.20E-13	PTTG1	-1.535258809	6.14E-49	TTK	-1.367397	4.66E-13	POLA1	-0.880600543	2.03E-22
RFC3	-1.0439	1.27E-15	RBL1	-1.247603049	8.54E-14				POLA2	-1.056102531	2.76E-07
RFC4	-0.756	0.00000153	RFC3	-1.471130255	5.97E-31				POLD1	-0.8571214	7.08E-13
RFC5	-0.6885	2.55E-08	RFC4	-0.751641191	1.14E-05				POLE	-0.884578266	2.97E-23
SGO1	-2.8274	2.53E-22	RFC5	-0.686187414	2.50E-06				POLE2	-1.99111447	3.95E-25
TICRR	-1.7747	2.81E-27	SGO1	-3.266046095	7.05E-24				PRIM1	-1.446557444	3.17E-11
TRIP13	-1.924	5.16E-47	TDP1	-0.600303399	5.45E-12				PRIM2	-0.675321158	1.49E-10
TTK	-2.700	9.97E-92	TICRR	-2.051485443	2.18E-33				PTTG1	-2.253531286	2.39E-86
			TRIP13	-2.151441151	2.88E-86				RBL1	-1.293480044	7.04E-21
			TTK	-3.565730345	2.95E-101				RFC2	-0.630456624	4.25E-10
									RFC3	-1.529500282	8.84E-36
									RFC4	-0.967229784	1.93E-17
									RFC5	-0.883297812	3.01E-15
									RPA3	-0.62335814	1.02E-06
									SGO1	-3.249692291	6.21E-40
									SMC1A	-0.60142879	8.57E-26
									TDP1	-0.73347864	2.40E-21
									TICRR	-2.47262082	6.32E-45
									TRIP13	-2.117954004	9.21E-141
									TTK	-3.191625323	1.02E-121

Supplementary Table S3. The impact of 2 or 5 Gy of IR on the cell cycle-related genes in HUVECs. Statistically significantly differentially expressed genes associated with cell cycle in irradiated HUVECs in comparison to their corresponding controls. Adjusted P-value < 0.05 and fold change (FC) > 1.5 (up-regulation) or FC < 0.67 (down-regulation) were considered as significance thresholds. *n* = 5. IR – irradiation.

IR with 2 Gy			IR with 5 Gy		
72 h vs 24 h after IR			72 h vs 24 h after IR		
Gene	log_2FC	Adjusted P-value	Gene	log_2FC	Adjusted P-value
AURKB	1.744739	1.41E-11	*AURKB*	0.780221	0.0488822
BUB1	1.676079	2.01E-27	*BUB1B*	0.780159	0.009926605
BUB1B	1.654532	6.78E-25	*CCNA2*	0.747508	0.000237499
CCNA2	1.660346	1.27E-40	*CCND2*	2.401424	7.78E-38
CCNB1	1.336363	3.31E-40	*CCNE1*	0.621735	0.000341976
CCNB2	1.351565	2.31E-14	*CDCA5*	0.713518	0.001354959
CCND2	1.921592	8.07E-27	*E2F1*	0.679856	0.01951901
CCNE1	0.838475	7.62E-08	*NDC80*	0.85965	0.001585443
CDC20	1.993271	8.65E-37	*PLK1*	0.648112	0.0036809
CDC25A	0.76653	0.000135789	*TTK*	0.684999	0.032792849
CDC25C	1.964437	1.09E-08			
CDC45	1.349567	3.10E-09			
CDC6	1.4032	7.07E-27			
CDCA5	1.445998	2.32E-19			
CDK1	1.293878	2.44E-16			
CDT1	1.244326	7.61E-08			
DBF4B	0.838373	0.00000424			
E2F1	1.627693	1.65E-15			
E2F2	1.66176	0.008239402			
ESCO2	1.790575	3.02E-10			
ESPL1	1.85528	2.41E-25			
FBXO5	0.735037	0.0000301			
FEN1	0.686991	0.000000194			
FOXM1	1.410089	2.48E-38			
KNL1	1.557735	2.91E-12			
MAD2L1	1.139093	3.71E-15			
MCM2	0.81823	0.00000314			
MCM4	0.970101	1.36E-24			
MYBL2	1.651611	5.57E-23			
NDC80	1.616568	1.03E-16			
NEIL3	1.52992	0.000000212			
ORC1	1.726822	7.75E-12			
ORC6	1.132887	2.21E-10			
PKMYT1	1.566751	1.21E-09			
PLK1	1.912427	5.66E-29			
POLA2	0.654796	0.022323855			
POLE2	0.792476	0.00356147			
PTTG1	0.816429	0.00000015			
RFC3	0.739536	0.005759172			
SGO1	1.974235	0.00000359			
TICRR	0.734703	0.005017163			
TRIP13	1.209348	5.53E-10			
TTK	1.617481	2.28E-12			

Supplementary Table S4. The impact of time after the IR with 2 or 5 Gy on cell cycle-related genes in HUVECs. Statistically significantly differentially expressed genes related to cell cycle in irradiated HUVECs, 72 h vs 24 h after IR. Adjusted P-value < 0.05 and fold change (FC) > 1.5 (up-regulation) or FC < 0.67 (down-regulation) were considered as significance thresholds. n = 5. IR – irradiation.

24 h after IR						72 h after IR					
2 Gy vs control			5 Gy vs control			2 Gy vs control			5 Gy vs control		
Gene	log_2FC	Adjusted P-value	Gene	log_2FC	Adjusted P-value	Gene	log_2FC	Adjusted P-value	Gene	log_2FC	Adjusted P-value
ADRB1	1.0712	0.012066751	*ADRB1*	0.949711	0.030253502	*CCND2*	1.317157	8.63E-15	*AGRN*	0.598249	1.93E-08
CDK1	-2.2756	3.77E-70	*CD36*	-0.83529	5.34E-11	*CDK1*	-1.15904	4.57E-30	*CCND2*	1.832944	5.96E-42
CLDN14	0.5984	0.009311982	*CDK1*	-2.96234	5.90E-115	*HGF*	-0.9898	0.000452584	*CD274*	0.593169	2.47E-16
HMMR	-2.8488	6.03E-91	*CLDN14*	0.817678	3.27E-05	*HMMR*	-1.35437	6.77E-26	*CDK1*	-2.7875	8.30E-194
MDM2	1.2203	1.75E-68	*EMP2*	-0.72734	1.61E-09	*IL1B*	0.604889	0.01625756	*CGN*	-3.01165	3.89E-05
TUBB4B	-0.6822	6.62E-51	*HMMR*	-3.31897	2.03E-98	*MDM2*	0.77424	5.13E-06	*CLDN14*	0.690567	0.000395655
			ICOSLG	0.90962	0.039284999	*TCF7*	-0.67152	0.046485839	*COL4A1*	0.606036	1.64E-17
			IKBKGP1	2.285301	0.007084417				*EMP2*	-0.94327	4.77E-16
			MDM2	1.557513	5.28E-84				*HGF*	-1.69972	7.06E-10
			MPP4	0.765551	0.004185172				*HLA-DPB1*	-0.84089	0.000498138
			PLCB2	0.696537	0.000414993				*HMMR*	-3.59262	2.08E-218
			PLK3	0.878722	2.30E-25				*ICOSLG*	1.139485	0.000130018
			SDC1	0.699244	1.67E-07				*LAMC2*	0.762489	6.35E-18
			SELP	-0.75035	7.78E-16				*MDM2*	1.017492	2.79E-44
			SORBS1	-0.89276	4.40E-10				*PARD6G*	0.939018	0.000870727
			TUBB4B	-0.60473	2.22E-11				*SELE*	0.618061	1.57E-06
									SORBS1	-0.59551	1.80E-06
									TUBA1B	-0.75007	1.12E-37
									TUBB4B	-0.79895	4.28E-33

Supplementary Table S5. Changes in the expression of ECM-associated genes after 2 or 5 Gy of IR in HUVECs. Statistically significantly differentially expressed genes associated with ECM in irradiated HUVECs compared to their corresponding controls. Adjusted P-value < 0.05 and fold change (FC) > 1.5 (up-regulation) or FC < 0.67 (down-regulation) were considered as significance thresholds. *n* = 5. IR – irradiation. ECM – extracellular matrix.

IR with 2 Gy			IR with 5 Gy		
72 h vs 24 h after IR			72 h vs 24 h after IR		
Gene	log_2FC	Adjusted P-value	Gene	log_2FC	Adjusted P-value
CCND2	1.921592	8.07E-27	*CCND2*	2.401424	7.78E-38
CD36	0.87341	1.05E-11	*CD36*	0.934579	3.51E-19
CDK1	1.293878	2.44E-16	*EMP2*	-0.60774	4.43E-06
HMMR	1.858725	2.29E-24	*ICAM1*	1.094083	2.80E-68
ICAM1	0.984602	1.15E-21	*IKBKGP1*	-2.05815	0.017056
IKBKGP1	-1.67143	0.03594931	*ITGA10*	-1.00053	1.90E-30
ITGA10	-0.93922	2.60E-24	*LAMC2*	1.68886	2.95E-62
LAMC2	1.499427	2.99E-118	*LLGL2*	-0.83697	0.001503
LLGL2	-1.19301	0.00000997	*MYLK2*	-0.76832	0.002886
MPP4	0.756257	0.00286183	*NEO1*	0.847391	3.63E-05
MYLK2	-0.8165	0.01588048	*PLCB2*	-0.59562	0.003214
NEO1	0.80446	3.84E-07	*SELE*	1.286093	1.12E-35
PLCB2	-0.66298	0.00890414	*SELP*	1.366922	1.56E-79
SELE	1.098239	3.56E-33			
SELP	1.05658	4.52E-24			

Supplementary Table S6. Time-dependent changes of ECM-associated genes after IR with 2 or 5 Gy in HUVECs. Statistically significantly differentially expressed genes associated with ECM in irradiated HUVECs, 72 h vs 24 h after IR. Adjusted P-value < 0.05 and fold change (FC) > 1.5 (up-regulation) or FC < 0.67 (down-regulation) were considered as significance thresholds. n = 5. IR – irradiation. ECM – extracellular matrix.

24 h after IR						72 h after IR					
2 Gy vs control			5 Gy vs control			2 Gy vs control			5 Gy vs control		
Gene	log_2FC	Adjusted P-value	Gene	log_2FC	Adjusted P-value	Gene	log_2FC	Adjusted P-value	Gene	log_2FC	Adjusted P-value
C5	-0.6043	0.049343797	*ACKR4*	-0.61469	2.20E-07	*CDCA3*	-1.09008	1.94E-15	*ACKR4*	-0.67654	9.68E-12
CD14	-0.9634	0.001450239	*AMPH*	-0.75717	0.000595171	*CXCL12*	0.612534	0.004311925	*C5*	-0.84233	0.000863736
CDCA3	-2.0401	1.58E-42	*BAMBI*	-0.59532	2.75E-05	*CXCL8*	0.871101	1.34E-08	*CARD16*	0.63013	1.51E-06
CXCL12	0.736	0.001605242	*BCL2L1*	0.687469	2.33E-23	*EDA2R*	1.288235	0.000732678	*CD55*	0.771882	3.92E-49
EDA2R	0.9996	0.006146979	*C5*	-0.71547	0.00858703	*FAS*	0.728913	0.000357009	*CDCA3*	-2.53584	2.75E-71
FAS	1.187	6.19E-26	*CD14*	-1.0849	0.001116763	*GDF15*	1.056797	1.34E-08	*CTSB*	0.732051	6.63E-75
GDF15	1.1836	4.79E-55	*CD36*	-0.83529	5.34E-11	*GDF3*	-0.95327	6.09E-13	*CXCL11*	0.595314	1.55E-05
GDF3	-0.7425	3.12E-09	*CDCA3*	-2.50692	2.04E-44	*IL1B*	0.604889	0.01625756	*CXCL12*	0.846451	8.13E-09
IL11	0.7741	0.004185231	*EDA2R*	0.969793	0.01687958	*MMP1*	0.732643	5.02E-24	*CXCL5*	-0.74344	6.86E-06
IL1B	0.8667	0.0000133	*F2RL3*	0.734472	0.005825602	*NOG*	0.882472	0.02081314	*CXCL8*	1.102786	3.06E-35
IL6	0.6018	0.003131212	*FAS*	1.467575	1.97E-43	*PIK3CG*	-0.81809	2.93E-05	*CXCR4*	0.724244	2.61E-10
LIF	0.6081	0.000477718	*GADD45A*	0.640903	1.47E-16	*PLA2G4C*	0.680596	3.37E-09	*EDA2R*	1.534436	1.92E-08
PIDD1	0.6052	8.39E-09	*GBP4*	-0.70432	0.017933486	*RASGRP3*	0.628253	5.29E-07	*F2RL3*	0.872598	2.33E-05
RBL1	-0.9199	1.20E-13	*GDF15*	1.720077	5.38E-82	*TNFRSF10C*	0.647982	6.12E-13	*FAS*	1.024804	3.27E-26
TNFSF4	0.9253	2.15E-44	*GDF3*	-0.90233	7.81E-08	*TNFSF4*	0.752039	9.83E-11	*GADD45A*	0.789441	4.86E-23
			GDF7	-0.70891	9.11E-10				*GDF15*	1.523855	1.76E-79
			IKBKGP1	2.285301	0.007084417				*GDF3*	-0.99246	4.34E-14
			IL12A	0.919481	0.001892221				*GSN*	0.655304	2.76E-17
			IL1B	0.678071	0.00206398				*HLA-DPB1*	-0.84089	0.000498138
			IL33	-0.6947	2.94E-19				*IL12A*	0.731125	0.012137496
			IL6	0.643825	0.001689589				*IL32*	0.650206	7.75E-12
			ITGAX	1.313249	0.005420613				*IL33*	-0.71835	1.34E-26
			LIF	0.64937	0.001354796				*JAG2*	0.591067	7.56E-16
			MMP1	0.62745	8.25E-13				*KITLG*	0.695967	8.28E-17
			P2RY1	-0.6826	0.000121688				*MME*	-0.98222	1.08E-16
			PIDD1	1.058744	3.80E-25				*MMP1*	1.149425	1.03E-110
			PIK3CG	-0.74844	2.50E-06				*NOG*	1.334458	6.73E-08
			PLAU	0.655465	4.08E-23				*PARP1*	-0.67246	5.28E-52
			PLCB2	0.696537	0.000414993				*PIDD1*	0.869275	1.20E-13
			RBL1	-1.2476	8.54E-14				*PIK3CG*	-1.31777	1.34E-21
			STING1	0.811453	2.32E-22				*PLA2G4C*	1.118336	5.56E-55
			TNFSF18	0.645891	0.000139807				*PLAU*	0.679912	2.08E-31
			TNFSF4	1.099259	4.95E-30				*RASGRP3*	0.876591	7.62E-20
			TRAF1	0.652146	1.57E-07				*RBL1*	-1.29348	7.04E-21
									SELE	0.618061	1.57E-06
									TANK	0.627734	2.76E-30
									TNFRSF10C	0.633939	3.62E-06
									TNFRSF4	0.92814	5.08E-05
									TNFSF4	1.054871	3.15E-69
									TNFSF9	0.714786	0.004949435
									TRAF1	0.762693	9.08E-12

Supplementary Table S7. The effect of 2 or 5 Gy IR on the immune response-associated genes in HUVECs. Statistically significantly differentially expressed genes associated with immune response in irradiated HUVECs (compared to their corresponding controls). Adjusted P-value < 0.05 and fold change (FC) > 1.5 (up-regulation) or FC < 0.67 (down-regulation) were considered as significance thresholds. *n* = 5. IR – irradiation.

IR with 2 Gy			IR with 5 Gy		
72 h vs 24 h after IR			72 h vs 24 h after IR		
Gene	log_2FC	Adjusted P-value	Gene	log_2FC	Adjusted P-value
C7	1.798292	0.000866	*C7*	1.478008	0.016008
CD36	0.87341	1.05E-11	*CD36*	0.934579	3.51E-19
CDCA3	1.214459	8.70E-10	*CD55*	0.753347	3.02E-23
CSF3	0.994632	0.022323	*CSF3*	0.918242	0.037291
CXCL1	0.916047	1.02E-20	*CTSB*	0.629424	2.05E-21
CXCL11	1.224528	8.08E-07	*CXCL1*	1.006664	7.39E-25
CXCL2	0.72173	1.3E-06	*CXCL11*	1.821114	2.59E-32
CXCL8	1.746534	1.19E-32	*CXCL12*	0.946711	8.40E-10
GDF7	0.827411	3.28E-15	*CXCL2*	0.725461	9.27E-06
ICAM1	0.984602	1.15E-21	*CXCL8*	2.132313	2.85E-88
IKBKGP1	-1.67143	0.035949	*GBP4*	0.70467	0.010919
IL1B	0.955689	6.90E-09	*GDF7*	1.048705	2.15E-15
IL1RL1	0.656396	6.50E-10	*ICAM1*	1.094083	2.80E-68
IL6	0.799206	6.64E-06	*IKBKGP1*	-2.05815	0.017056
LIF	0.712168	2.96E-06	*IL1B*	0.989769	1.87E-10
MYLK2	-0.8165	0.01588	*IL6*	0.682665	0.000101
NEO1	0.80446	3.84E-07	*ITGAX*	-1.04784	0.014036
PLA2G4C	0.606416	1.16E-07	*MYLK2*	-0.76832	0.002886
PLAT	0.663431	6.15E-18	*NEO1*	0.847391	3.63E-05
PLAU	0.806628	5.51E-26	*PLA2G4C*	0.93475	1.00E-26
PLCB2	-0.66298	0.008904	*PLAU*	0.775868	1.90E-30
SELE	1.098239	3.56E-33	*PLCB2*	-0.59562	0.003214
TNFRSF25	-0.69154	1.3E-06	*SELE*	1.286093	1.12E-35
TNFSF9	0.732968	0.021465	*TNFRSF1B*	-0.63685	9.38E-18
			TNFRSF25	-0.66685	1.87E-06
			TNFRSF4	0.616665	0.031524

Supplementary Table S8. Time-dependent changes of immune response-related genes after IR with 2 or 5 Gy in HUVECs. Statistically significantly differentially expressed genes associated with immune response in irradiated HUVECs, 72 h vs 24 h after IR. Adjusted P-value < 0.05 and fold change (FC) > 1.5 (up-regulation) or FC < 0.67 (down-regulation) were considered as significance thresholds. $n = 5$. IR – irradiation.

	15 Gy - 48 h vs Control		
	Pathway	NES	Adjusted P-value
Up-regulated	HALLMARK_INTERFERON_GAMMA_RESPONSE	2.755437805	0.000588998
	HALLMARK_INTERFERON_ALPHA_RESPONSE	2.675417555	0.000588998
	HALLMARK_ALLOGRAFT_REJECTION	2.17398615	0.000588998
	KEGG_CYTOKINE_CYTOKINE_RECEPTOR_INTERACTION	2.1149331	0.000588998
	HALLMARK_TNFA_SIGNALING_VIA_NFKB	2.091384374	0.000588998
	REACTOME_EXTRACELLULAR_MATRIX_ORGANIZATION	2.075976016	0.000588998
	HALLMARK_INFLAMMATORY_RESPONSE	2.073554426	0.000588998
	REACTOME_INTERFERON_GAMMA_SIGNALING	2.06819669	0.00100533
	REACTOME_IMMUNOREGULATORY_INTERACTIONS_BETWEEN_A_LYMPHOID_AND_A_NON_LYMPHOID_CELL	2.054895952	0.002544873
	HALLMARK_IL6_JAK_STAT3_SIGNALING	2.034462421	0.00100533
	HALLMARK_COAGULATION	2.018891424	0.00100533
	REACTOME_INTERFERON_ALPHA_BETA_SIGNALING	2.016276001	0.001369767
	REACTOME_ELASTIC_FIBRE_FORMATION	1.943484675	0.00514143
	REACTOME_MOLECULES_ASSOCIATED_WITH_ELASTIC_FIBRES	1.928410706	0.006175765
	HALLMARK_EPITHELIAL_MESENCHYMAL_TRANSITION	1.919508147	0.000588998
Down-regulated	HALLMARK_E2F_TARGETS	-2.939406568	0.000588998
	HALLMARK_G2M_CHECKPOINT	-2.758211559	0.000588998
	REACTOME_MITOTIC_SPINDLE_CHECKPOINT	-2.626792565	0.000588998
	REACTOME_RESOLUTION_OF_SISTER_CHROMATID_COHESION	-2.624404133	0.000588998
	REACTOME_MITOTIC_PROMETAPHASE	-2.561979411	0.000588998
	REACTOME_CELL_CYCLE_CHECKPOINTS	-2.561950361	0.000588998
	REACTOME_CELL_CYCLE_MITOTIC	-2.511754004	0.000588998
	REACTOME_CELL_CYCLE	-2.488136123	0.000588998
	REACTOME_MITOTIC_METAPHASE_AND_ANAPHASE	-2.477215964	0.000588998
	REACTOME_SEPARATION_OF_SISTER_CHROMATIDS	-2.423305603	0.000588998
	REACTOME_CHROMOSOME_MAINTENANCE	-2.386254417	0.000588998
	REACTOME_M_PHASE	-2.362457127	0.000588998
	HALLMARK_MYC_TARGETS_V1	-2.350195191	0.000588998
	REACTOME_RHO_GTPASES_ACTIVATE_FORMINS	-2.341525631	0.000588998
	REACTOME_REPRODUCTION	-2.287957535	0.000588998

Supplementary Table S9. Top 15 significantly up- and down-regulated pathways in TECs *in vivo* at 48 h after 15 Gy of IR. As described by Kaeppler et. al. described, a single dose of 15 Gy of IR was delivered to the tumors while mice anesthetized with isoflurane were restrained in a lead shield, leaving only the tumors exposed to the IR [7,27]. Significantly enriched pathways identified by GSEA analysis. Publicly available dataset of murine colon carcinomas MC38 (GSE168481). n = 5. IR - irradiation. GSEA – Gene Set Enrichment Analysis. NES – Normalized Enrichment Score.
